# Functionalization of 3-chloroformylcoumarin to coumarin Schiff bases using reusable catalyst: an approach to molecular docking and biological studies

**DOI:** 10.1098/rsos.172416

**Published:** 2018-05-02

**Authors:** Suresh S. Kumbar, Kallappa M. Hosamani, Gangadhar C. Gouripur, Shrinivas D. Joshi

**Affiliations:** 1 Department of Studies in Chemistry, Karnatak University, Dharwad, 580003, Karnataka, India; 2 P. G. Department of Biotechnology and Microbiology, Karnatak University, Dharwad, 580003, Karnataka, India; 3 Novel Drug Design and Discovery Laboratory, Department of Pharmaceutical Chemistry, S.E.T.'s College of Pharmacy, Sangolli Rayanna Nagar, Dharwad 580002, Karnataka, India

**Keywords:** silica sulfuric acid, Schiff's bases, molecular docking, *in vitro* anti-tubercular, antimicrobial, cytotoxicity study

## Abstract

Recently, heterogeneous catalysts have been explored eximiously in the synthesis of heterocyclic compounds. Therefore, here we used solid-supported heterogeneous silica sulfuric acid as a catalyst for the synthesis of Schiff's base of 3-chloroformylcoumarin in view of simplified procedure, reusability and acceptable efficiency, which are required in organic synthesis. An efficient and facile methodology is preferred for synthesis of a class of chromeno-3-substituted derivatives (**1a–1l**) with good yields. The molecular docking results showed excellent binding interactions with the *Mycobacterium tuberculosis* InhA-D148G mutant (PDB: 4DQU). The same biomolecules were screened for their *in vitro* anti-tubercular activity against the *M.tb* H37Rv strain and antimicrobial studies. Physico-chemistry, toxicity prediction with IC50 value and bioactivity score were also calculated for title compounds. Most active compounds were further tested for cytotoxicity studies and exhibited low-level cytotoxicity against Vero cells. The suggested conjugates are promising lead compounds for the subsequent investigation in search of new anti-tubercular agents. All the conjugates were obtained within the range and followed the Lipinski rule of 5, indicating more ‘drug-like’ nature.

## 1. Introduction

Owing to the growing concern of chemicals and their impact on the environment, cleaner reaction conditions in chemical synthetic procedures are needed to be incorporated. The intensive endorsement to maintain greenness requires us to avoid secondary substances (e.g. organic solvents, additional toxic reagents), to stop the overproduction of waste and minimize the consumption of energy [1]. Recently, the use of heteropolyacid catalysts, especially reusable solid catalysts, has gained a leading role in organic synthesis due to their environmental and economic considerations, and industrial utilization [2]. The high catalytic activity, moisture sensitivity, reusability and notably low cost makes solid-supported reagents attractive substitutes to conventional Lewis acids [3,4]. A number of synthetic schemes have been described for the synthesis of various heterocycles including the condensation of aldehydes with substituted aromatic amines [5–8]. However, most of these reports have some flaws such as lengthened reaction times, use of overpriced toxic solvents/chemicals, harsh reaction conditions, occurrence of several side products and/or lesser yields. Although the chemical utilization of solid-supported reagents for organic synthesis has been well explored, there are relatively few literature reports on the use of silica sulfuric acid (SSA) [9–14].

**Scheme 1.**
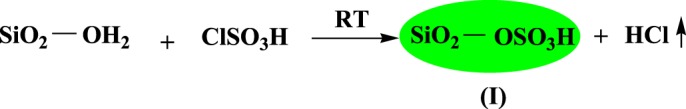
Preparation of silica sulfuric acid.

SSA is an acid catalyst, simply prepared by using chlorosulfonic acid with silica gel at ambient temperature (scheme 1) [15]. Easy handling, low price, efficiency, recoverability and reusability make SSA eco-friendly and a synthetically acceptable catalyst. We recognized that SSA would be an excellent proton entity compared to all the reported acidic reagents or acidic resins like polystyrene sulfonic acid and Nafion-H [16] under heterogeneous reaction conditions. SSA enhances reactivity and selectivity and has synthetic applicability in organic reactions such as oxidation [17], formation of carbon–carbon bonds [18,19], cycloaddition, [20] protection–deprotection steps [21,22], esterification [23] and the synthesis of heterocycles [24]. SSA is easier to handle than other acidic reagents and can be readily taken out of the reaction mixture by simple filtration. Additionally, it is recyclable and may be applicable on an industrial scale in pharmaceuticals. Therefore, we expanded the applications of SSA as a new versatile heterogeneous acid as a reusable catalyst for the title compound synthesis.

On the contrary, tuberculosis (TB) is a foremost communicable and infectious airborne, contagious, deadly disease caused by the pathogenic bacterium *Mycobacterium tuberculosis* (*M. tb*) of the ‘tuberculosis complex’ [25], which had evolved sometime around the seventh millennia BC, but has been making recent appearances and this malady has not yet been able to be completely eradicated. Robert Koch identified *M. tb* as the ‘white plague’ in 1882 [26]. It has attained epidemic proportions over wide geographical regions in the world. According to the World Health Organization, 10–12 million new TB cases are detected every year and has been estimated to be a major cause of death (2–3 million/year) [27,28]. A high rate of new cases of TB infections and deaths in HIV-positive patients gives a compelling view of AIDS in developing countries [29]. TB treatment lacks new medications, even after the development of potent drug therapy for *M. tb* treatment. The current drugs available in the market for standard TB treatment are limited by certain formidable challenges including lengthy treatment of 6–9 months, multiple drug regimens and chemical side effects [30]. The problem is becoming worse by increasing resistance to standard available drugs and synergy of this disease with HIV and infections in immunocompromised patients [31]. Therefore, latent TB patients are more affected by HIV because HIV weakens their immune system and makes them much more prone to developing active TB. Patients who are co-infected with HIV have an up to 800 times more chance of becoming infectious with active TB [32]. The constant increase in multidrug-resistant strains of *M. tb* has additionally contributed to the demand for new anti-TB drugs. However, over a decade no new TB drugs have been introduced into clinical use. Drugs active against resistant forms of TB are less effective and more toxic, and need to be taken for an extended regimen of up to 18 months [33]. This has inspired us for new efforts to find potent anti-TB drug candidates, which comprises developing pipelines for drug discovery and enhancing the ease of synthesis, in particular trying to implement new treatments that can considerably shorten the duration of effective therapy, which would improve patient compliance and survivability with novel mechanisms of action [34–36].

**Figure 1.**
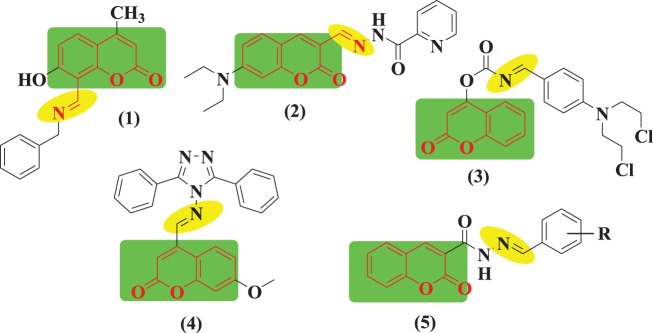
Some coumarin Schiff base derivatives.

Coumarin is an oxygenated heterocycle; structurally it is the least complex component and forms a huge group of conjugates belonging to the flavonoid class of plant secondary metabolites, which have a special role in nature with their wide spectrum of biological applications [37]. They have attracted increased attention in recent decades for their diverse pharmacological properties. Among all properties, their cytotoxic effects have been most extensively studied [38,39]. Naturally occurring calanolide A and B are conjugates of coumarins that have evoked appreciable interest for their dual activity against TB and HIV infections [40]. Some antibiotics *viz*. novobiocin, clorobiocin and coumermycin A1 are composed of a coumarin nucleus. It has been established that coumarin heterocycles are an important component in designing a new class of structural biomolecules for medicinal applications. It is evident from earlier reports that substitution of coumarin at all positions except in the one and two positions with various functionalities has led to potent anti-TB activity. The alkyl substituents at the third position may change to aryl and heterocyclic groups, and evinced excellent anti-TB activity. Especially, coumarin bearing substitutions at the third and fourth positions was found to be more active due to conjugation [41]. Cardoso *et al*. reported a series of *N*-benzylidene-2-oxo-2H-chromene-3-carbohydrazides as substituents at the third position of coumarin, and examined their non-infected cell viabilities and anti-TB performance against *M. tb*, and the results are compared to pyrazinamide (PZA), which is a first-line anti-TB drug. Compound (**5**) in figure 1 exhibited an antimycobacterial activity at 50 mg ml^−1^ and was found more active than the reference drug PZA [42].

**Figure 2.**
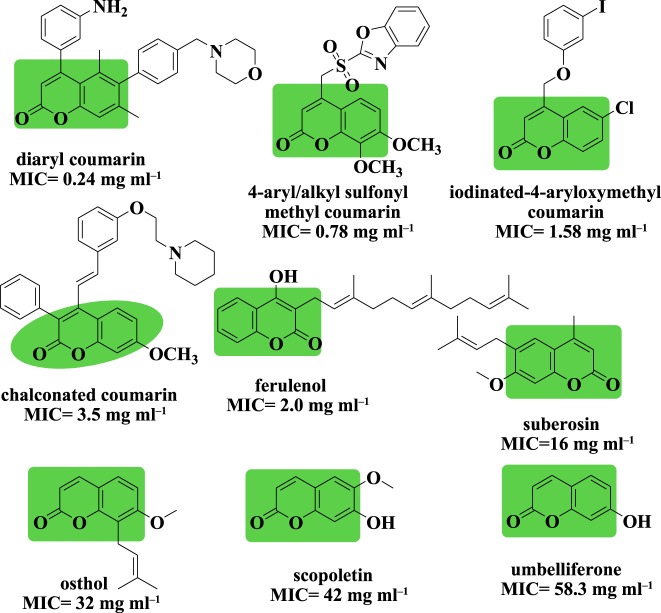
Naturally occurring and synthetic coumarins exhibiting anti-TB properties.

Imines with a hydroxyl moiety of aromatic heterocycles are of particular interest. It is known that bioactive compounds bearing an imine group, with variation in substitutions, are important structures because of their biological properties such as anticancer and anti-tubercular activities [43]. The reaction of heterocyclic aldehydes and substituted anilines resulted in substituted heterocyclic Schiff bases. This type of building block is able to coordinate with metal ions and hydrogen bonds, and accept protons at the cellular level. Along with enzymatic interactions and receptors, it also controls physico-chemical properties of desired molecules to exhibit a broad spectrum of bioactivity. Hence looking into the biological significance of coumarins, particularly in the field of TB, we anticipate that coumarins could be a good starting point for the development of new lead anti-tubercular drugs. Figure 2 presents the structures of some potent coumarin scaffolds exhibiting anti-tubercular properties.

**Figure 3.**
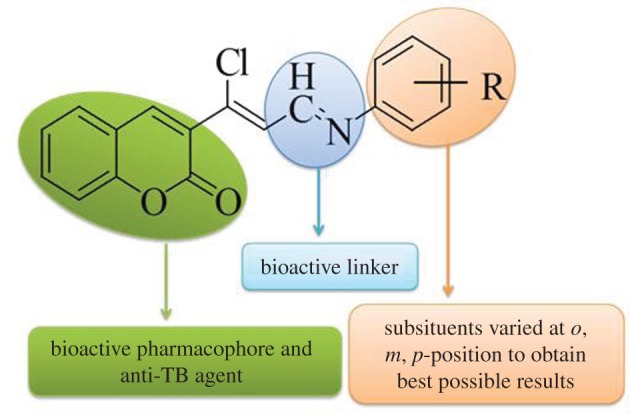
The hypothetical interaction model designed for coumarin compounds.

Today, the Lipinski rule of 5 (RO5) is widely used by medicinal chemists worldwide to evaluate not only the absorption of compounds but also specific drug similarity [44]. Hence, considering the highlights of RO5, we designed coumarin compounds and analysed their physico-chemical properties set by RO5, drug-likeness, toxicity prediction with IC50 value and bioactivity scores. It was found that all the derived conjugates were obtained within the frame of RO5. Considering the diverse biological and physico-chemical properties of coumarin compounds, there has been a growing interest in the synthesis of coumarin Schiff bases. It was thought that these two active pharmacophores, i.e. coumarin and aromatic amines, linked together through an imine bond would generate novel molecular templates with the most potential to exhibit anti-tubercular properties. Hence, our present strategy is to prepare coumarin derivatives having all these subunits in one structural frame (figure 3) which might exhibit enhanced synergistic effect and activity. The broad spectrum of their biological activity makes them a promising subject for the synthesis of new derivatives to identify lead bioactive compounds. The ongoing work uses the (*M. tb*) *H_37_Rv* strain to screen the anti-tubercular property of synthesized coumarin drugs.

Thus, in the view of the current interest in environmental protection, we extended environmentally benign heterogeneous acid catalysts for simple synthesis of coumarinyl Schiff bases. These derivatives (**1a–1l**) are examined for *in vitro* anti-tubercular, antibacterial and antifungal activities.

## 2. Material and methods

All the chemicals were purchased commercially. The open capillary method was used for detection of melting points, which are uncorrected. IR spectra were recorded using a Nicolet 5700 FT-IR (Nicolet, Madison, WI, USA) with KBr discs for all derivatives. ^1^H and ^13^C NMR spectra were recorded with a Bruker 400 MHz spectrometer using CDCl_3_/dimethylsulfoxide (DMSO) as solvents and are reported as δ values (ppm). Mass spectra were recorded with a Shimadzu GCMSQP2010S. The elemental analyses were carried out with a Hereaus CHN rapid analyser. Thin-layer chromatography (TLC) was used during the reaction for monitoring the progress of the reaction.

### 2.1. Chemistry

**Scheme 2.**
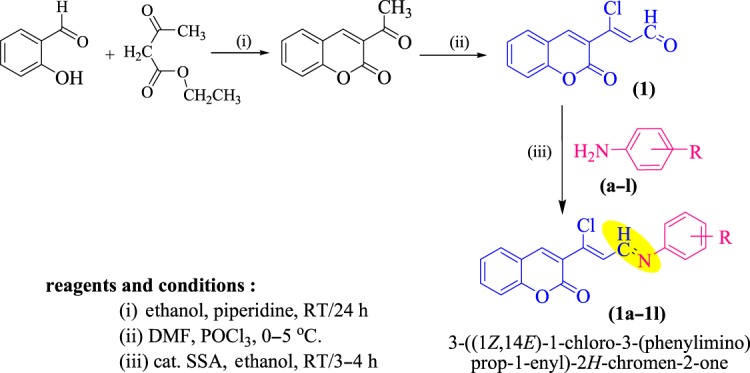
Synthesis of novel coumarin Schiff bases (1a–1l).

The conjugate of coumarinyl Schiff bases (1a–1l) was synthesized by using SSA. At first, 3-formylchlorocoumarin (1) was obtained efficiently in good yields by the Vilsmeir–Hack formylation reaction of 3-acetylcoumarin. Further, 3-formylchlorocoumarin (1) (1 mmol) with substituted anilines (a–l) (1 mmol) in 10 ml ethanol and a catalytic amount of SSA at room temperature (RT) afforded 3-((1*Z*,14*E*)-1-chloro-3-(phenylimino)prop-1-enyl)-2H-chromen-2-one (1a–1l) under the conventional method, as is stated in scheme 2.

**Scheme 3.**
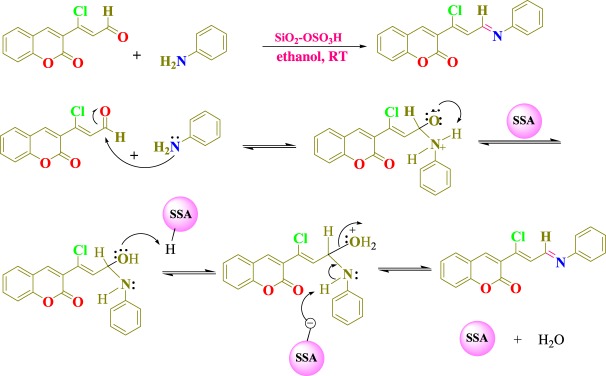
Proposed reaction pathway for the formation of compound 1a.

It was observed that use of the SSA approach proved to be extremely fast, providing good to excellent yields (58–78%). The results are stated in table 1. The most evident advancement in the synthesis was the speed at which the reaction proceeded; the reactions were completed within 3 to 4 h. Optimization was also featured in different polar protic and aprotic solvents (table 2). The reaction carried out in acetonitrile gave the product in lesser yield at RT (entries 1 and 8). Use of solvent ethanol yielded the product with a shorter reaction time of 3 h at RT with an overall yield of 78% (entry 2). Further, dioxane in 6 h yielded the product with 38% yield (entry 4); in the case of tetrahydrofuran (THF) traces of the product were obtained, whereas no product was obtained in dimethylformamide (DMF), DMSO and acetone solvents even after a longer reaction time of 12 h. In the absence of SSA, even after longer time the reaction did not initiate at room temperature, resulting in incompletion of the reaction (entry 9). The reaction was carried out with silica alone, and a longer time for the completion of the reaction was noted. This may be due to the less acidic property of silica (entry 10). Here we generally saw evidence of imide formation in instances with excellent yields. This allowed us to state a mechanism for the formation of 1a using SSA as shown in scheme 3. Initially, the attack of electrons of aniline on the aldehydic carbon of coumarin takes place. In the consecutive step, the protonation occurs from SSA, forming itself as a nucleophile in the pool of reaction mixture. The moment water is eliminated from the reaction mass, the nucleophilic SSA abstracts protons from nitrogen and gains stability by the formation of a double bond between C and N. To the best of our knowledge, this represents the first time that SSA has been used to catalyse the direct Schiff's bases of 3-formylchlorocoumarins and substituted anilines under normal reaction conditions. However, the fact that there was an example where SSA proved to be a catalyst suitable for the cleavage of the carbon–nitrogen double bond of Schiff's bases in dioxane under conventional heating at reflux conditions raised a concern about our postulate that the overall conversion is merely a direct formation of double bond that happens when a catalytic amount is used at RT, rather than a cleavage of the double bond [45]. For example, the excess amounts of SSA at reflux is a condition which limits the applicability of such protocols. Therefore, the development of a new method which is free from such a problem is necessary. To understand the mechanistic insight further, several experiments were repeated with lesser SSA equivalents. Here we generally observed the formation of imide and in instances where a good yield was observed.

**Table 1.** Analytical data of synthesized coumarin derivatives (1a–1l).

products	R	yield (%)	time (min)	melting point (°C)
1a	H	78	180	165–167
1b	p-Cl	62	210	193–195
1c	p-Br	61	190	182–184
1d	p-OH	67	195	198–200
1e	p-OCH_3_	62	210	205–208
1f	p-CH_3_	71	240	202–204
1g	2,6-dimethyl	58	220	188–190
1h	m-Cl	68	210	197–200
1i	m-Br	69	190	178–181
1j	m-OH	62	195	184–186
1k	m-OCH_3_	59	200	208–210
1l	m-CH_3_	73	225	212–214

**Table 2.** Optimization of solvent selection for the synthesis of coumarin compounds.

entry	solvent	SSA (eq.)	time (h)	temperature (°C)	yield (%)
1	acetonitrile	1.0	4	25	35
2	ethanol	1.0	3	25	78
3	DMF	1.0	12	25	nil
4	dioxane	1.0	6	25	38
5	THF	1.0	12	25	trace
6	DMSO	1.0	12	25	nil
7	acetone	1.0	12	25	nil
8	acetonitrile	2.0	12	40	42
9	ethanol	0.0	12	25	nil
10	ethanol	Si^a^	12	25	45

^a^Only silica is used to perform this experiment.

## 3. Results and discussion

Chromeno-3-substituted Schiff's base hybrids were confirmed by spectroscopic analysis, as in the case of compound 3-((1*Z*,14*E*)-1-chloro-3-(phenylimino)prop-1-enyl)-2H-chromen-2-one (**1a**).

The IR spectrum exhibited a band at 1723 cm^−1^ assignable to lactone carbonyl stretching, whereas the –C=N stretching appears at 1600 cm^−1^.

Depiction of the product was further confirmed by the ^1^H NMR spectrum, wherein one singlet corresponding to C4-H of coumarin appeared in the downfield region at δ 9.179 ppm. Two doublets corresponding to C13-H and C5-H of coumarin resonate at δ 8.670 and 8.055 ppm (*J* = 7.6 Hz); adjacent to it another sharp triplet was observed at δ 7.765 ppm (*J* = 8.4 Hz), which is assigned to the C7-H of the coumarin ring; next to it another doublet was observed at δ 7.553 ppm (*J* = 7.2 Hz), which corresponds to C8-H of coumarin. The C6-H of coumarin resonate as a triplet at δ 7.430 ppm (*J *= 7.6 Hz and 7.2 Hz), whereas the C12-H resonate as a doublet at δ 6.417 ppm. The remaining aromatic protons resonate in their expected aromatic region at δ 7.186–7.343 ppm.

^13^C NMR provides additional support for the structure of 1a. The carbon of the lactone carbonyl (–C=O) resonates at δ 161.656 ppm. The –C9 carbon adjacent to coumarin oxygen resonates at 161.055 ppm and the carbon of –C=N– resonates at δ 154.342 ppm. –C4 and –C3 carbons of coumarin resonate at δ 149.833 and 129.56 ppm, respectively, and –C11 and –C12 carbons resonate at δ 126.54 and 116.54 ppm, respectively. The remaining carbons resonated at δ 114.19–153.059 ppm, which is in agreement with their expected values.

The molecular ion peak at 309 [M]^+^ in the EI-MS proved to be further support to the architecture of 1a. The bond between the C11 and the Cl atom is polar, and significant fragmentations take place on these carbons, giving a peak at *m/z* 274. The mass peak at *m/z* 44 is due to elimination of CO_2_. All the remaining coumarin derivatives furnished satisfactory spectroscopic and analytical data. All data are in accordance with the assigned structures and are stated in the experimental section.

### 3.1. Biological evaluation

The coumarin derivatives were examined for the potential *in vitro* anti-tubercular properties against the *M. tb H_37_Rv* strain by the Microplate Alamar Blue Assay (MABA) [46]. The most active derivatives found were tested for their cytotoxicity against Vero cells by the MTT [47] assay. Further, title compounds were tested for their antifungal and antibacterial properties by the disc diffusion method. The molecular docking study was used to find out the interactions of small coumarin-derived molecules and receptors in proteins. The crystal structure of the *M. tb* InhA-D148G mutant (PDB ID: 4DQU) in complex with NADH (2.45 Å X-ray resolutions) was used for this study. Physico-chemical, *in silico* toxicity prediction with IC50 value and bioactivity score were also calculated for the title compounds.

#### 3.1.1. Anti-tubercular screening

The title (**1a–1l**) compounds were initially examined for *in vitro* anti-TB activity at a concentration of 6.25 µg ml^−1^ against the *M. tb H_37_Rv* strain in BACTEC 12B medium using the MABA. Compounds exhibiting inhibition ≥ 90% in the initial evaluation were tested at below 6.25 µg ml^−1^ using twofold dilution in the range of 3.12–0.2 µg ml^−1^ to find out the actual minimum inhibitory concentration (MIC). The anti-tubercular results are presented in table 3. In the primary screening, most of the compounds (**1b, 1c, 1e**, **1h, 1i** and **1k**) displayed 90–100% inhibition. At the second level, two derivatives (**1e** and **1k**) showed inhibition with MIC < 0.2** **µg ml^−1^ and four compounds **1b, 1c, 1h** and **1i** with MIC < 2** **µg ml^−1^, when checked with standard isoniazid (MIC 0.02** **µg ml^−1^). From table 3, it is observed that the electron-releasing –OCH_3_-substituted compounds (**1e**) and (**1k**) have shown more significant inhibitory activity with an MIC of 0.05 and 0.19** **µg ml^−1^, respectively. The activity increased with the change in the position of –OCH_3_ group in the following sequence p-OCH_3 _> m-OCH_3_. The halogens –Cl and –Br, substituents at the *para* and *meta* position of the phenyl ring (**1b, 1c, 1h** and **1i**), exhibited MIC in the range of 1.21–3.12** **µg ml^−1^, while the –CH_3_-substituted compounds were found to be unreactive.

**Figure 4.**
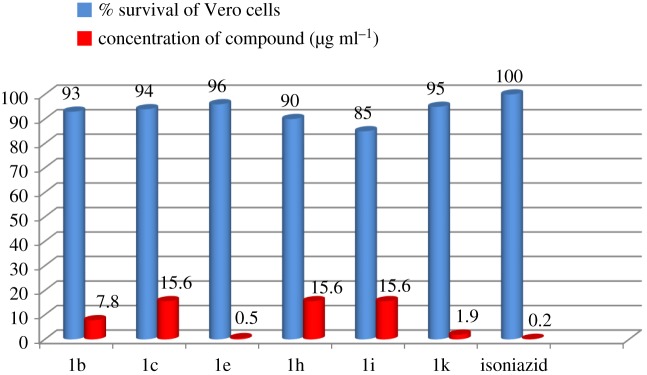
Comparison between per cent survival of Vero cells at a concentration of the compound 10 times that of the actual MIC value (µg ml^−1^).

**Table 3.** Results of *in vitro* anti-TB screening against *M. tb* H_37_Rv and cytotoxicity assay against Vero cells (n.d., not determined). The most active compounds are marked in bold**.**

compound	R	% inhibition at a concentration of 6.25 µg ml^−1^	MIC^a^ (µg ml^−1^)	% survival of Vero cells at conc. (10 × MIC)^b^
1a	−*H*	78	n.d.	n.d.
**1b**	***p-Cl***	**93**	**0.78**	**79**
**1c**	***p-Br***	**94**	**1.56**	**72**
1d	*p-OH*	67	n.d.	n.d.
**1e**	***p-OCH_3_***	**96**	**0.05**	**93**
1f	*p-CH_3_*	78	n.d.	n.d.
1g	−*2,6-dimethyl*	66	n.d.	n.d.
**1h**	***m-Cl***	**90**	**1.56**	**65**
**1i**	***m-Br***	**85**	**1.56**	**62**
1j	*m-OH*	75	n.d.	n.d.
**1k**	***m-OCH_3_***	**95**	**0.19**	**92**
1l	*m-CH_3_*	72	n.d.	n.d.
**isoniazid**	**—**	**100**	**0.02**	**100**

^a^Minimum inhibitory concentration against the *H_37_R*v strain of *M. tuberculosis* (µg ml^−1^).

^b^Compound is considered toxic if it causes over 50% inhibition of normal cells at a concentration 10-fold higher than its MIC value.

#### 3.1.2. Cytotoxic studies

To obtain insights into potential toxicities of the highly active 3-((1*Z*,14*E*)-1-chloro-3-(phenylimino)prop-1-enyl)-2H-chromen-2-one hybrids, compounds (**1b, 1c, 1e, 1h, 1h** and **1k**) were examined for their cytotoxicity against Vero cells, at concentrations 10 times their actual MIC value. A compound is considered toxic if it causes more than 50% inhibition of normal cells at a concentration 10-fold higher than its MIC [48]. The results are stated in table 3.

From the results, it is concluded that most of the screened coumarin derivatives have been found to exhibit a good safety profile. Among the tested compounds, –OCH_3_ substituents (**1e** and **1k**) showed the highest safety profile with a greater than 90% survival rate of Vero cells, indicating good selectivity, as shown in figure 4.

#### 3.1.3. *In vitro* antifungal and antibacterial screening

The title compounds (1a–1l) were screened for antifungal activity against pathogenic fungal strains *C. albicans* (ATCC 14053)*, C. utilis* (MTCC 183)*, T. rubrum* (MTCC 296)*, T. mentagrophytes* (MTCC 7687)*, A. niger* (ATCC 16888)*, A. flavus* (ATCC 9643D-2)*, A. fumigates* (ATCC 4609D-2) and *T. glabrata* (MTCC 3602). A 5% DMSO solution is used to dissolve compounds, and the disc diffusion method is used to determine the antimicrobial activity in terms of the zone of inhibition [49]. All incubations were performed in triplicate. Among the tested compounds, the compounds **1b, 1c, 1e, 1h, 1i** and **1k** have emerged as active against all tested fungal strains, and exhibited an excellent zone of inhibition, bearing –Cl, –Br and –OCH_3_ groups varied at the *para* and *meta* positions of the phenyl ring, whereas **1f** and **1l** with methyl groups showed moderate activity. **1a, 1d, 1g,** and **1j** showed much less activity against some fungal strains and were found inactive. The results are tabulated in table 4 and are expressed in terms of the diameter of growth of the inhibition zone (mm).

**Table 4.** *In vitro* antifungal activity of (1a–1l) derivatives. Fungal strains: *Candida albicans, Candida utilis, Trichophyton rubrum, Trichophyton mentagrophytes, Aspergillus niger, Aspergillus flavus, Aspergillus fumigates* and *Torulopsis glabrata*. Standard antibiotic disc: ampicillin (10 mcg). (−) negative results. Values are mean ± s.e.m. All incubations were performed in triplicate. The best values for each compound are provided in bold.

	diameter of growth of the inhibition zone (mm)							
compound code	*C. albicans* ATCC 14053	*C. utilis* MTCC 183	*T. rubrum* MTCC 296	*T. mentagraphytes* MTCC 7687	*A. niger* ATCC 16888	*A. flavus* ATCC 9643D-2	*A. fumigatus* ATCC 4609D-2	*T. glabrata* MTCC 3602
1a	6.3 ± 0.4	6.8 ± 0.1	—	8.0 ± 0.3	—	—	7.2 ± 0.4	—
1b	18.1 ± 0.1	—	16.2 ± 0.0	10.3 ± 0.0	19.00 ± 0.1	16.1 ± 1.2	17.00 ± 0.7	14.4 ± 0.5
1c	21.0 ± 0.2	13.8 ± 0.3	16.8 ± 0.0	13.3 ± 0.4	12.2 ± 0.06	14.00 ± 0.1	—	13.4 ± 0.5
1d	—	—	10.3 ± 0.3	—	6.4	—	7.4 ± 0.1	—
**1e**	—	**14.4 **± **0.0**	**15.5 **± **0.0**	**12.0 **± **02**	**13.8 **± **1.2**	**14.0 **± **0.4**	**12.1 **± **0.6**	**16.2 **± **0.3**
1f	12.2 ± 0.2	12.8 ± 0.1	10.4 ± 0.2	13.2 ± 0.0	9.0 ± 0.2	10.2 ± 0.0	—	10.3 ± 0.3
1g	9.6 ± 1.2	6.8 ± 0.3	—	11.7 ± 0.2	—	—	8.2 ± 0.1	—
1h	16.2 ± 0.0	10.0 ± 0.6	13.3 ± 0.2	14.9 ± 0.5	15.0 ± 0.0	12.3 ± 1.0	—	13.2 ± 04
1i	17.5 ± 0.4	11.8 ± 0.1	13.6 ± 0.8	12.2 ± 0.4	—	13.1 ± 0.0	12.8 ± 0.0	14.2 ± 0.1
1j	—	—	8.6 ± 0.0	—	9.4 ± 0.3	—	6.3 ± 0.2	—
**1k**	**15.3 **± **1.0**	**12.6 **± **0.0**	—	**12.5 **± **0.0**	**13.4 **± **0.6**	**12.2 **± **0.0**	**11.6 **± **0.7**	**14.8 **± **0.1**
1l	10.2 ± 0.2	11.3 ± 0.0	8.0 ± 0.3	10.6 ± 0.1	6.6 ± 0.0	8.4 ± 0.8	—	9.2 ± 1.0
ampicillin	22.2 ± 0.3	20.5 ± 0.2	21.4 ± 0.2	22.5 ± 0.0	21.2 ± 0.3	18.5 ± 1.2	20.4 ± 1.0	21.6 ± 0.3

Compounds (**1a–1l**) were also examined for their antibacterial activity against pathogenic bacterial strains *S. aureus* (ATCC 29413)*, B. subtilis* (NCIB 8057)*, E. coli* (ATCC 25992)*, P. aeruginosa* (NCIB 8295)*, B. cereus* (ATCC 11778)*, K. pneumonia* (ATCC 10031)*, S. typhi (*14028) and *C. botulinum* (ATCC 443) by the disc diffusion method, and all incubations and tests were performed in triplicate. The obtained results are almost mimicking the results which are obtained for antifungal studies. The antibacterial results revealed that the compounds **1b, 1c, 1e, 1h, 1i** and **1k** turned out to be active against all tested bacterial strains and exhibited an excellent zone of inhibition bearing –Cl, –Br and –OMe groups varied at the *para* and *meta* positions, and **1f** and **1l** showed good activity, whereas **1a, 1d, 1 g** and **1j** were found to be less active against some bacterial strains or inactive. The results are tabulated in table 5 and are expressed in terms of the diameter of growth of the inhibition zone (in mm).

**Table 5.** *In vitro* antibacterial activity of (1a–1l) derivatives. Bacterial strains: *Staphylococcus aureus*, *Bacillus subtilis*, *Escherichia coli*, *Pseudomonas aeruginosa*, *Bacillus cereus*, *Klebsiella pneumonia*, *Salmonella typhi* and *Clostridium botulinum*. Standard antibiotic disc: penicillin and streptomycin (10 mcg), (−) negative results. Values are mean ± s.e.m. All incubations were performed in triplicate. The best values for each compound are provided in bold.

	diameter of growth of the inhibition zone (mm)							
compound code	*S. aureus* ATCC 29413	*B. subtilis* NCIB 8057	*E. coli* ATCC 25992	*P. aeruginosa* NCIB 8295	*B. cereus* ATCC 11778	*K. pneumonia* ATCC 10031	*S. typhi* 14028	*C. botulinum* ATCC 443
1a	5.3 ± 0.33	—	—	7.6 ± 0.33	7.3 ± 0.16	6.3 ± 0.00	—	10.5 ± 0.28
1b	14.8 ± 0.4	16.2 ± 0.0	12.3 ± 0.8	—	14.8 ± 0.3	11.4 ± 0.2	10.5 ± 0.2	14.3 ± 0.6
1c	10.6 ± 0.5	12.8 ± 1.2	11.0 ± 0.1	12.2 ± 0.3	14.0 ± 0.0	—	11.5 ± 0.6	13.0 ± 0.05
1d	—	6.4 ± 0.8	—	5.8 ± 0.4	—	8.2 ± 0.3	—	—
**1e**	**14.6 **±** 0.1**	**—**	**14.2 **± **0.06**	**10.0 **± **0.3**	**13.4 **± **0.6**	**11.04 **± **0.0**	**14.6 ± 0.2**	**16.8 **± **0.1**
1f	9.0 ± 0.4	10.5 ± 0.2	9.2 ± 0.2	8.6 ± 0.1	—	11.04 ± 0.3	—	10.6 ± 0.2
1g	6.4 ± 0.01	—	—	6.2 ± 0.0	7.3 ± 0.0	—	—	7.8 ± 0.02
1h	—	9.6 ± 0.0	11.8 ± 0.0	13.8 ± 0.3	10.00 ± 0.2	13.02 ± 0.3	12.4 ± 0.6	12.3 ± 0.9
1i	13.9 ± 0.3	13.0 ± 0.06	10.00 ± 0.8	—	10.5 ± 0.0	12.00 ± 1.2	8.3 ± 0.1	14.3 ± 0.0
1j	—	5.6 ± 0.3	—	8.6 ± 0.6	6.8 ± 02	6.3 ± 00	—	—
**1k**	**13.2 **± **0.0**	**10.8 **± **0.0**	**11.6 **± **0.3**	**—**	**10.3 **± **0.0**	**12.8 **± **0.0**	**12.0 ± 0.6**	**15.0 **± **0.1**
1l	7.4 ± 0.0	8.5 ± 0.0	10.3 ± 0.2	7.6 ± 0.8	—	8.8 ± 1.0	—	8.2 ± 0.0
penicillin	12.5 ± 0.0	19.6 ± 03	22.2 ± 0.0	20.0 ± 0.3	20.3 ± 1.2	24.3 ± 0.1	20.0 ± 0.6	—
streptomycin	20.0 ± 1.2	12. 0 ± 0.4	20.3 ± 0.8	20.4 ± 0.0	—	12.6 ± 0.5	20.4 ± 0.3	19.6 ± 0.0

The enhanced antifungal and antibacterial activities of the compounds **1b, 1c, 1e, 1h, 1i** and **1k** could be attributed by the presence of –OCH_3_, and halogen **(**–Cl and –Br) groups varied at the phenyl ring. However, based on this promising observation, it is premature to arrive at the conclusion on the structure–activity aspect of these molecules, and further evaluation is needed to use them for clinical use.

### 3.2. Molecular docking studies

**Figure 5.**
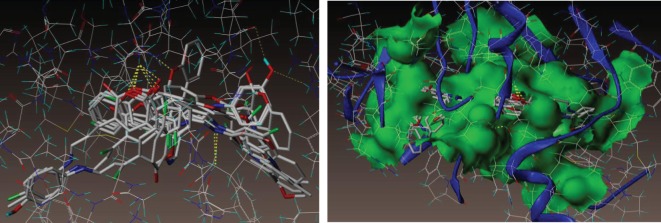
Docked view of all compounds into the active site of the enzyme 4DQU.

The coumarin compounds were docked with the *M. tb* InhA-D148G mutant (PDB ID: 4DQU) using the surflex-dock program of sybyl-X 2.0. The synthesized inhibitors were docked into active sites of 4DQU protein, as shown in figure 5. The predicted binding interaction energies of inhibitors are stated in table 6. The docking studies revealed that all the coumarin inhibitors have been found to exhibit potential binding interactions into active sites and to inhibit the activities of *M. tb,* showing very good docking scores.

**Table 6.** Surflex docking score (kcal mol^−1^) of the chromene derivatives (potential of mean force, PMF). Data for compounds showing higher C score value are in bold.

compounds	C score^a^	crash score^b^	polar score^c^	D score^d^	PMF score^e^	G score^f^	chem score^g^
1a	6.83	−1.10	1.05	−108.646	5.536	−168.419	−27.374
1b	5.88	−1.16	0.21	−122.068	−9.315	−164.857	−31.901
1c	5.22	−1.24	0.00	−113.756	−19.898	−167.179	−30.478
**1d**	**6**.**82**	**−1**.**54**	**1**.**10**	**−116**.**886**	**−5**.**681**	**−165**.**504**	**−28**.**743**
**1e**	**7**.**29**	**−1**.**71**	**0**.**00**	**−126**.**825**	**−20**.**380**	**−185**.**269**	**−30**.**855**
1f	6.28	−0.92	0.00	−107.641	−18.601	−152.499	−30.541
1g	6.75	−2.49	0.00	−123.300	−12.031	−212.371	−34.665
1h	5.53	−0.97	1.06	−96.994	13.947	−140.737	−25.646
1i	5.94	−1.93	1.23	−120.551	−7.124	−157.632	−31.556
**1j**	**7**.**58**	**−1**.**24**	**0**.**00**	**−116**.**753**	**−12**.**270**	**−179**.**032**	**−31**.**601**
**1k**	**7**.**24**	**−1**.**75**	**0**.**67**	**−120**.**512**	**−18**.**302**	**−168**.**669**	**−29**.**523**
1l	6.46	−1.65	1.59	−120.066	8.647	−194.331	−25.538

^a^C score (consensus score) integrates a number of popular scoring functions for ranking the affinity of ligands bound to the active site of a receptor and reports the output of the total score.

^b^Crash score revealing the inappropriate penetration into the binding site. Crash scores close to 0 are favourable. Negative numbers indicate penetration.

^c^Polar indicating the contribution of the polar interactions to the total score. The polar score may be useful for excluding docking results that make no hydrogen bonds.

^d^D score for charge and van der Waals interactions between the protein and the ligand.

^e^PMF score indicating the Helmholtz free energies of interactions for protein–ligand atom pairs.

^f^G score showing hydrogen bonding, complex (ligand–protein) and internal (ligand–ligand) energies.

^g^Chem score points for H-bonding, lipophilic contact and rotational entropy, along with an intercept term.

**Figure 6.**
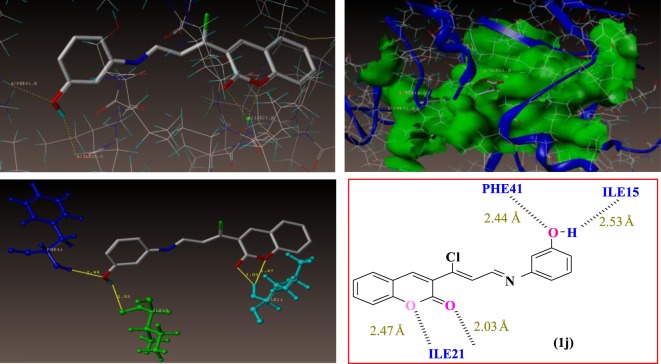
Binding interactions of compound 1j into the active site of 4DQU.

The docking results suggest that the compound **1j** (m-OH) has shown the least binding energies, with more hydrogen bond interaction than other derivatives as illustrated in figure 6. The compound **1j** has shown excellent binding interaction with 4DQU and exhibited a better inhibition constant and also makes four hydrogen bond interactions with the active site of the enzyme. These four interactions were found with different amino acids such as ILE21, PHE41 and ILE15. Among those interactions were one from carbonyl oxygen of chromene ring with hydrogen of 21st amino acid, i.e. isoleucine and a distance of 2.03 Å (H-ILE21, 2.03 Å) and oxygen of chromene ring with hydrogens of ILE21 (H-ILE21, 2.47 Å), oxygen atom of hydroxyl group present on the *meta* position of phenyl ring makes hydrogen bonding interaction with hydrogen of PHE41 with distance of 2.44 Å (H-PHE41, 2.44 Å) and remaining hydrogen bonding interactions arising from the hydrogen atom of hydroxyl group present on the third position of phenyl ring with oxygen of ILE15 (O=C-ILE15, 2.53 Å).

**Figure 7.**
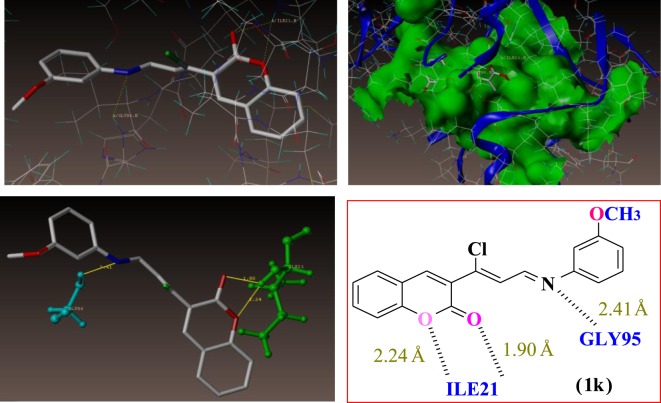
Binding interactions of compound 1k into the active site of 4DQU.

As illustrated in figure 7 compound 1 k (m-OCH_3_) makes three hydrogen bond interactions: two interactions were of the oxygen atom of the carbonyl group of the chromene ring at distance 1.90 Å with hydrogens of ILE21 (H-ILE21, 1.90 Å) and that of the chromene ring oxygen atom with hydrogens of ILE21 (H-ILE21, 2.24 Å), and the third was that of the nitrogen atom of the C=N group with hydrogen of GLY96 (CH=N–H-GLY96, 2.41 Å).

**Figure 8.**
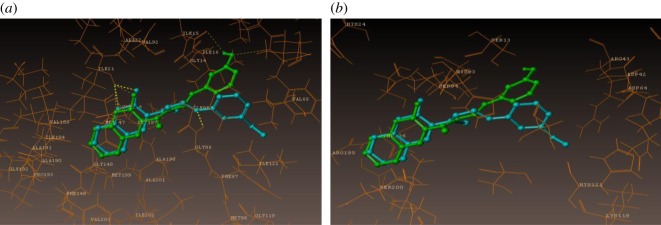
Represents the compounds 1j and 1k surrounded by hydrophobic and hydrophilic amino acids. (*a*) Hydrophilic and (*b*) hydrophobic amino acids surrounding compounds 1j (green colour) and 1k (cyan colour).

Molecular docking studies revealed that the compact skeleton of coumarin is the basic reason of how it holds strong contacts with the important amino acid (hydrophobic and hydrophilic) side chains inside the active site as well as the adjoining sites of the enzyme (figure 8*a,b*), thus preventing its pro-tubercular active role. It has been concluded that hydroxyl and methoxy substituted Schiff bases were accommodated more in the predicted allosteric active site than in the metal-binding site. The docking results are presented in table 6.

### 3.3. Physico-chemical properties

The theoretical calculation of absorption, distribution, metabolism, excretion and toxicity properties for synthesized compounds are calculated and compared with RO5. This is expressed as the octanol/water partition coefficient called logP; other theoretical calculations such as topological polar surface area, number of hydrogen bond acceptors and number of hydrogen bond donors were also performed. All these physico-chemical properties of synthesized compounds are stated in table 7. All the coumarin compounds showed good agreement and followed RO5, indicating more **‘**drug-like’ nature, and none of the compounds violate RO5.

**Table 7.** Drug-likeness property (RO5) of compounds (1a–1j). HBA, number of hydrogen bond acceptors (n-ON); HBD, number of hydrogen bond donors (n-OHNH); LogP, logarithm of partition coefficient between *n*-octanol and water (miLogP); TPSA, topological polar surface area; GPCR, G-protein-coupled receptors**.**

	Lipinski's parameters							bioactivity score					
compound	HBA	HBD	LogP	violations	TPSA	molar volume (A^3^)	drug likeness	GPCR ligand	ion channel modulator	kinase inhibitor	nuclear receptor ligand	protease inhibitor	enzyme inhibitor
**1a**	3	0	4.33	0	42.58	264.21	**0**.**41**	−0.59	−0.78	−0.73	−0.38	−0.64	−0.34
**1b**	3	0	5.01	1	42.58	277.74	**0**.**38**	−0.55	−0.75	−0.70	−0.37	−0.62	−0.36
**1c**	3	0	5.14	1	42.58	282.09	**0**.**28**	−0.66	−0.82	−0.73	−0.47	−0.71	−0.41
**1d**	4	0	3.85	0	62.80	272.23	**0**.**23**	−0.50	−0.70	−0.64	−0.20	−0.57	−0.27
**1e**	4	0	4.38	0	51.81	289.75	−0.09	−0.57	−0.79	−0.69	−0.34	−0.60	−0.37
**1f**	3	0	4.78	0	42.58	280.77	**0**.**26**	−0.60	−0.82	−0.73	−0.38	−0.65	−0.40
**1g**	3	0	5.13	1	42.58	297.33	**0**.**23**	−0.60	−0.80	−0.72	−0.39	−0.60	−0.36
**1h**	3	0	4.98	0	42.58	277.74	**0**.**08**	−0.56	−0.75	−0.69	−0.38	−0.66	−0.38
**1i**	3	0	5.11	1	42.58	282.09	**0**.**07**	−0.69	−0.83	−0.70	−0.52	−0.72	−0.43
**1j**	4	1	3.83	0	62.80	272.23	−0.13	−0.52	−0.71	−0.63	−0.23	−0.59	−0.29
**1k**	4	0	4.36	0	51.81	289.75	**0**.**08**	−0.58	−0.80	−0.68	−0.37	−0.62	−0.39
**1l**	3	0	4.75	0	42.58	280.77	**0**.**07**	−0.61	−0.84	−0.73	−0.38	−0.65	−0.41

#### 3.3.1. *In silico* toxicity prediction

The best way to calculate the toxic effect of drug molecules is developing animal models, which is ideal. Based on compounds of known drug candidates and their toxicity by toxic fragments or chemical structure, the Web server ProTox estimates rodent oral toxicity [50]. This compares the similarity of synthesized compounds which will be loaded in a server with a database of compounds having known toxicity previously, recognizing the toxic fragments of coumarin compounds and possible toxicity targets. The server requires only the two-dimensional structure for which prediction is to be carried out.

The predicted LD50 (lethal dose) with a range of 190–1950 mg kg^−1^ for all the compounds is summarized in table 8. As claimed by the developer's limits, all the synthesized compounds come under the classes 4 and 5 toxicity category and there are no toxic fragments present. This toxicity prediction study reveals that coumarin compounds can act as the lead compounds for further detailed investigations. Based on the investigation of *in silico* toxicology, all the compounds have shown median LD50 values in the range of 190–1950 mg kg^−1^. Compounds **1d** and **1j** showed an LD50 value of 190** **mg kg^−1^ bearing a hydroxyl group, whereas compounds **1b** and **1h** showed an LD50 value of 820** **mg kg^−1^ with a chlorine group, and remaining compounds exhibited a wide range of LD50 values of 1600–1950 mg kg^−1^. All the targeted compounds belong to toxicity classes 4 and 5, and none of them have toxic fragments. The predicted results are tabulated in table 8.

**Table 8.** Oral toxicity prediction results of coumarin Schiff base (1a–1l) derivatives.

compound code	predicted LD50 (mg kg^−1^)	predicted toxicity class	average similarity (%)	prediction accuracy (%)	toxic fragments
1a	1600	5	38.57	23	nil
1b	820	4	37.64	23	nil
1c	1680	4	36.68	23	nil
1d	190	4	39.1	23	nil
1e	1700	5	38.49	23	nil
1f	1600	5	38.28	23	nil
1g	1600	5	38.68	23	nil
1h	820	4	37.04	23	nil
1i	1680	4	36.11	23	nil
1j	190	4	38.58	23	nil
1k	1950	5	37.50	23	nil
1l	1600	5	37.64	23	nil

### 3.4. Preliminary structure–activity relationship study

Although the number of compounds examined here is limited, a few key features regarding structural requirements for these 3-((1*Z*,14*E*)-1-chloro-3-(substituted phenylimino)prop-1-enyl)-2H-chromen-2-one (**1a–1l**) to exert their anti-tubercular properties may be observed. Our initial strategy was to determine the key substructure necessary for bioactivity such as azomethane group (enhances both the pharmacokinetic and pharmacodynamic properties of biomolecules) and coumarin (active pharmacophore, which allows its derivatives to readily interact with a variety of enzymes and receptors in organisms). Further essential substituents like (R=CH_3_ (electron-donating), –OCH_3_ (electron-releasing), and –Br, –Cl (halogens)) groups were substituted at the *para* and *meta* positions of the anilines to get the excellent results as represented in the designed hypothetical interaction module (figure 3).

The results revealed the assumptions for preliminary structure–activity relationships (SAR) stated as follows: all the results confirm that, in a series of compounds having –OCH_3_ substituents (1e and 1k) influencing the anti-tubercular activity significantly, particularly –OCH_3_ at the *para* position (1e) was found to be the most active *in vitro,* exhibiting an MIC of 0.05** **µg ml^−1^. A slight change in the position of –OCH_3_ from *para* to *meta* (1k) lowers the activity with an MIC of 0.19** **µg ml^−1^. The halogenated derivation at the *para* position exposed the second line of activity by (1b) and (1c), exhibiting comparatively good activity with MIC values of 1.21 and 1.56** **µg ml^−1^, respectively; the change in the position of the halogen from *para* to *meta* (1h) and (1i) slightly lowers the activity with MIC values of 1.56 and 2.81** **µg ml^−1^, respectively, considering that the electron-donating methyl substituents (1f) and (1l) are found to be inactive against the *M. tb* test.

From the overall analysis, it is concluded that derivatives bearing the –OCH_3_ substituent have been found to possess more significant anti-tubercular properties than derivatives with –Cl, –Br, –OH and –CH_3_, substituents on the aniline ring are found to be moderate, while the –CH_3_ substituents were found to be inactive against the *M. tb* strain. Thus, it is evident that the substituents at the *para* position of the aniline ring are found to be much more active and potent when compared with the same substituent at the *meta* position. In fact the *ortho* substituents were found to diminish or reduce the overall activity of the inhibitors. Hence, it can also be hypothesized that steric hindrance might have also played a promising role in influencing the activity of the *ortho*-substituted derivatives. The attribution from the preliminary SAR analysis has led to the determination of some key structural requirements for the 3-((1*Z*,14*E*)-1-chloro-3-(substituted phenylimino)prop-1-enyl)-2H-chromen-2-one hybrids to exert their anti-TB property, which provides insights into further structural modifications.

## 4. Conclusion

In summary, a simple and efficient protocol for the synthesis of coumarin Schiff base derivatives using SSA was accomplished. In the primary screening for anti-TB, most of the compounds (**1b, 1c, 1e, 1h, 1i** and **1k**) displayed about 90–100% inhibition. In the secondary level, two compounds (**1e** and **1k**) inhibited *M. tb* with MIC < 0.2** **µg ml^−1^, which is in good agreement with molecular docking results. Four compounds (**1b, 1c, 1h** and **1i**) were found with MIC < 2** **µg ml^−1^, when compared with isoniazid. Further, among the tested compounds, –OCH_3_ substituents (**1e** and **1k**) exhibited an excellent safety profile with over 90% survival rate of Vero cells, indicating good selectivity. Compounds with –Cl, –Br and –OMe groups varied at the *para* and *meta* positions showed high potency, in that methyl substituents exhibited good results towards both antifungal and antibacterial activity. Molecular docking studies provided the binding insights consistent with the acceptor and the donor of the title compounds. These studies have been used to correlate and support our experimental results. Also we found that compounds (**1j** and **1k**) with –OH and –OCH_3_-substituted coumarin molecules make hydrogen bonding interactions, which were explored in hydrophobic binding residues, with the electron-donating group in hydrophilic residues probably beneficial for enhancing the binding interactions. The toxicity prediction study reveals that coumarin compounds can act as the lead compounds for further investigations and potent applications of pharmacological interest. From the overall findings, this study suggests that all the potentiality is because of the oxygenated coumarin heterocycle, which later was enhanced by condensing substituted anilines and could be used as a drug to inhibit the occurrence of TB. However, further detailed investigation of coumarin is needed for the exploration of its potency, which can provide lead candidates for drug development in the treatment of such diseases.

## 5. Experimental set-up

### 5.1. Synthesis of 3-formylchlorocoumarin (1)

3-Formylchlorocoumarin was obtained efficiently by the Vilsmeir–Hack formylation reaction of 3-acetylcoumarin under cold conditions.

### 5.2. General procedure for the preparation compounds (1a–1l).

A mixture of substituted aromatic amines (0.01 mol) and (2*Z*)-3-chloro-3-(2-oxo-2H-chromen-3-yl)acrylaldehyde (0.01 mol) was diluted in 10 ml of ethanol; to this mixture a catalytic amount of SSA was added, and this was stirred at RT for about 3–4 h. The progress of the reaction was monitored by TLC. After completion of the reaction, the catalyst was filtered off and the reaction mixture was quenched onto crushed ice; the solid product obtained was filtered and washed with water and recrystallized from ethanol.

#### 5.2.1. 3-((1Z,14E)-1-chloro-3-(phenylimino)prop-1-enyl)-2H-chromen-2-one (1a)

Light yellow solid; Mp 165–167°C; IR (KBr) (*v*_max_/cm^−1^): 1723 (C=O of coumarin), 1600 (C=N of azomethane) cm^−1^; ^1^H NMR (400 MHz, CDCl_3_, δ ppm): 6.417 (d, 1H), 7.186–7.343 (m, Ar-H, 5H), 7.43(t, 1H, *J *= 7.2 Hz), 7.553 (d, 1H, *J *= 7.6 Hz), 7.765(t, 1H, *J *= 8.4 Hz), 8.055 (d, 1H, *J *= 6.4 Hz), 8.670(d, 1H), 9.179 (s, 1H); ^13^C NMR (100 MHz, CDCl_3_, δ ppm): 114.921, 116.54, 121.444, 121.727, 124.797, 126.54, 128.097, 128.12, 129.402, 129.565, 133.094, 133.455, 136.488, 149.833, 153.509, 154.342, 161.053, 161.656; GC-MS: 309 [M]^+^; Anal. calcd for C_18_H_12_ClNO_2_: Found: C, 69.85; H, 3.93; N, 4.47%.

#### 5.2.2. 3-((1Z,14E)-3-(4-chlorophenylimino)-1-chloroprop-1-enyl)-2H-chromen-2-one (1b)

Light yellow solid; Mp 193–195°C; IR (KBr) (*v*_max_/cm^−1^): 1721 (C=O of coumarin), 1625 (C=N of azomethane) cm^−1^; ^1^H NMR (400 MHz, CDCl_3_, δ ppm): 6.480 (d, 1H), 7.224–7.379 (m, Ar-H, 4H), 7.481 (t, 1H, *J *= 7.2 Hz, 6.8 Hz), 7.647 (d, 1H, *J *= 7.2 Hz), 7.748 (t, 1H, *J *= 7.2 Hz, 6.8 Hz), 8.042 (d, 1H, *J *= 8.8 Hz), 8.594 (d, 1H, *J *= 9.6 Hz), 9.15 (s, 1H); ^13^C NMR (100 MHz, CDCl_3_, δ ppm): 115.402, 116.105, 116.762, 124.197, 125.030, 129.504, 129.537, 131.105, 131.762, 136.369, 138.199, 141.787, 148.702, 154.192, 155.155, 160.656; GC-MS: 344 [M]^+^; Anal. calcd for C_18_H_11_Cl_2_NO_2_: Found: C, 62.85; H, 3.24; N, 4.05%.

#### 5.2.3. 3-((1Z,14E)-3-(4-bromophenylimino)-1-chloroprop-1-enyl)-2H-chromen-2-one (1c)

Buff colour solid; Mp 182–184°C; IR (KBr) (*v*_max_/cm^−1^): 1719 (C=O of coumarin), 1626 (C=N of azomethane) cm^−1^; ^1^H NMR (400 MHz, CDCl_3_, δ ppm): 6.850 (d, 1H, *J *= 6.8 Hz), 7.071–7.213 (m, Ar-H, 4H), 7.279 (d, 1H, *J *= 7.2 Hz), 7.499 (t, 1H, *J *= 7.6 Hz), 7.550 (t, 1H, *J *= 8.4 Hz), 8.265 (d, 1H, *J *= 9.6 Hz), 9.133 (s, 1H); ^13^C NMR (100 MHz, CDCl_3_, δ ppm): 98.76, 101.71, 107.05, 108.62, 112.67, 116.73, 116.93, 121.52, 130.13, 142.26, 147.46, 148.54, 151.63, 152.04, 153.45, 160.44; GC-MS: 388 [M]^+^; Anal. calcd for C_18_H_11_BrClNO_2_: Found: C, 55.58; H, 2.93; N, 3.57%.

#### 5.2.4. 3-((1Z,14E)-3-(4-hydroxyphenylimino)-1-chloroprop-1-enyl)-2H-chromen-2-one (1d)

Light yellow solid; Mp 198–200°C; IR (KBr) (*v*_max_/cm^−1^): 3424 (OH), 1713 (C=O of coumarin), 1646 (C=N of azomethane) cm^−1^; ^1^H NMR (400 MHz, CDCl_3_, δ ppm): 6.17 (s, 1H), 6.63 (d, *J* = 5.5 Hz, 1H), 6.91 (d, *J* = 14.2 Hz, 2H), 7.17–7.57 (m, 5H), 7.93 (d, *J* = 7.2 Hz, 1H), 8.16 (d, *J* = 7.6 Hz, 1H), 8.41 (s, 1H); ^13^C NMR (100 MHz, CDCl_3_, δ ppm): 110.78, 111.07, 112.73, 116.37, 116.60, 118.42, 125.73, 125.84, 127.30, 141.85, 144.93, 152.30, 152.62, 154.69, 161.35, 164.68; ESI-MS: 325 [M]^+^; Anal. calcd for C_18_H_12_ClNO_3_: Found: C, 66.43; H, 3.73; N, 4.27%.

#### 5.2.5. 3-((1Z,14E)-3-(4-methoxyphenylimino)-1-chloroprop-1-enyl)-2H-chromen-2-one (1e)

Light yellow crystals; Mp 205–208°C; IR (KBr) (*v*_max_/cm^−1^): 1716 (C=O of coumarin), 1629 (C=N of azomethane) cm^−1^; ^1^H NMR (400 MHz, CDCl_3_, δ ppm): 3.592 (s, 3H), 6.20 (d, 1H), 7.102–7.163 (m, Ar-H, 4H), 7.379 (t, *J *= 7.6 and 8.8 Hz, 1H), 7.473 (d, *J *= 8.4 Hz, 1H), 7.705 (t, *J *= 7.6 Hz, 1H), 8.051 (d, *J *= 4.4 Hz, 1H), 8.816 (d, *J *= 7.6 Hz, 1H), 9.179 (s, 1H); ^13^C NMR (100 MHz, CDCl_3_, δ ppm): 68.19, 100.54, 103.48, 108.83, 110.40, 114.45, 118.51, 118.71, 123.30, 131.91, 133.22, 144.03, 149.24, 150.31, 153.41, 155.23, 162.27; GC-MS: 339 [M]^+^; Anal. calcd for C_19_H_14_ClNO_3_: Found: C, 67.15; H, 4.13; N, 4.77%.

#### 5.2.6. 3-((1Z,14E)-3-(*p*-tolylimino)-1-chloroprop-1-enyl)-2H-chromen-2-one (1f)

Light yellow solid; Mp 202–204°C; IR (KBr) (*v*_max_/cm^−1^): 1715 (C=O of coumarin), 1626 (C=N of azomethane) cm^−1^; ^1^H NMR (400 MHz, CDCl_3_, δ ppm): 2.618 (s, 3H), 6.577 (d, 2 Hz, 1H), 6.987–7.038 (m, Ar-H, 2H), 7.338–7.458 (m, 3H), 7.623 (t, *J *= 7.6 and 8 Hz, 2H), 8.167 (d, *J *= 9.2 Hz, 1H), 8.415 (s, 1H), 9.01 (s, 1H); ^13^C NMR (100 MHz, CDCl_3_, δ ppm): 31.24, 110.43, 111.49, 113.17, 118.02, 126.17, 126.35, 129.13, 130.39, 131.56, 135.06, 141.49, 151.49, 154.08, 154.81, 159.65, 164.86; GC-MS: 323 [M]^+^; Anal. calcd for C_19_H_14_ClNO_2­_: Found: C, 70.45; H, 4.35; N, 4.37%.

#### 5.2.7. 3-((1Z,14E)-3-(2,6-dimethylphenylimino)-1-chloroprop-1-enyl)-2H-chromen-2-one (1g)

White solid; Mp 188–190°C; IR (KBr) (*v*_max_/cm^−1^): 1720 (C=O of coumarin), 1634 (C=N of azomethane) cm^−1^; ^1^H NMR (400 MHz, CDCl_3_, δ ppm): 2.41 (s, 6H), 6.57 (d, *J* = 2.3 Hz, 1H), 6.72 (d, *J* = 8.2 Hz, 1H), 7.27–7.36 (m, 3H), 7.946 (d, 1H), 8.15 (d, *J* = 5.6 Hz 1H), 8.615 (s, 1H); ^13^C NMR (100 MHz, CDCl_3_, δ ppm): 22.14, 109.51, 110.57, 112.25, 117.10, 125.24, 125.42, 128.33, 129.47, 130.64, 134.13, 140.56, 150.57, 153.16, 153.88, 158.72, 163.94; GC-MS: 337 [M]^+^; Anal. calcd for C_20_H_16_ClNO_2_: Found: C, 71.15; H, 4.73; N, 4.17%.

#### 5.2.8. 3-((1Z,14E)-3-(3-chlorophenylimino)-1-chloroprop-1-enyl)-2H-chromen-2-one (1h)

Light yellow solid; Mp 198–200°C; IR (KBr) (*v*_max_/cm^−1^): 1720 (C=O of coumarin), 1644 (C=N of azomethane) cm^−1^; ^1^H NMR (400 MHz, CDCl_3_, δ ppm): 6.39 (s, 1H), 6.60 (s, 1H), 6.78 (d, *J* = 7.6 Hz, 1H), 7.18–7.122 (m, 3H), 7.45–7.47 (m, 2H), 7.95 (d, *J* = 7.6 Hz, 1H), 8.21 (d, *J* = 8 Hz,1H), 8.76 (s, 1H); ^13^C NMR (100 MHz, CDCl_3_, δ ppm): 109.68, 109.97, 111.63, 115.27, 115.50, 117.32, 124.63, 124.74, 126.20, 140.75, 143.83, 143.90, 151.21, 151.52, 153.59, 160.25, 160.29, 163.59; GC-MS: 344 [M]^+^; Anal. calcd for C_18_H_11_Cl_2_NO_2_: Found: C, 62.85; H, 3.23; N, 4.09%.

#### 5.2.9. 3-((1Z,14E)-3-(3-bromophenylimino)-1-chloroprop-1-enyl)-2H-chromen-2-one (1i)

Buff colour solid; Mp 178–181°C; IR (KBr) (*v*_max_/cm^−1^): 1714 (C=O of coumarin), 1628 (C=N of azomethane) cm^−1^; ^1^H NMR (400 MHz, CDCl_3_, δ ppm): 6.63 (s, 1H), 7.18–7.122 (m, 2H), 7.45–7.47 (m, 2H), 7.63 (d, *J* = 7.6 Hz, 1H), 7.67 (s, 1H), 7.78 (d, *J* = 6.8 Hz, 1H), 7.95 (d, *J* = 7.6 Hz, 1H), 8.14 (d, *J* = 8 Hz,1H), 8.69 (s, 1H); ^13^C NMR (100 MHz, CDCl_3_, δ ppm): 108.10, 108.39, 110.05, 113.69, 113.92, 115.74, 123.05, 123.16, 124.62, 139.17, 142.25, 142.32, 149.63, 149.94, 152.07, 158.67, 158.71, 162.01; GC-MS: 388 [M]^+^; Anal. calcd for C_18_H_11_BrClNO_2_: Found: C, 55.65; H, 2.93; N, 3.57%.

#### 5.2.10. 3-((1Z,14E)-3-(3-hydroxyphenylimino)-1-chloroprop-1-enyl)-2H-chromen-2-one (1j)

Light yellow solid; Mp 184–186°C; IR (KBr) (*v*_max_/cm^−1^): 3427 (OH), 1714 (C=O of coumarin), 1624 (C=N of azomethane) cm^−1^; ^1^H NMR (400 MHz, CDCl_3_, δ ppm): 6.628 (d, *J* = 5.6 Hz, 1H), 6.89 (s, 1H), 7.168 (m, 5H), 7.436 (m, 3H), 8.161 (d, *J* = 7.6 Hz, 1H), 8.589 (s, 1H); ^13^C NMR (100 MHz, CDCl_3_, δ ppm): 104.28, 112.33, 112.75, 114.15, 114.52, 121.52, 123.84, 125.88, 129.33, 129.38, 129.71, 133.98, 135.79, 148.51, 152.90, 156.42, 161.21, 162.36; GC-MS: 325 [M]^+^; Anal. calcd for C_18_H_12_ClNO_3_: Found: C, 66.45; H, 3.73; N, 4.27%.

#### 5.2.11. 3-((1Z,14E)-3-(3-methoxyphenylimino)-1-chloroprop-1-enyl)-2H-chromen-2-one (1k)

Light yellow solid; Mp 208–210°C; IR (KBr) (*v*_max_/cm^−1^): 1711 (C=O of coumarin), 160 (C=N of azomethane) cm^−1^; ^1^H NMR (400 MHz, CDCl_3_, δ ppm): 3.825 (s, 3H), 6.428 (d, *J* = 2.4 Hz, 1H), 6.58 (d, *J* = 2.0 Hz, 1H), 6.636 (s, 1H), 6.732 (d, *J* = 8.2 Hz, 1H), 7.26–7.38 (m, 5H), 8.17 (d, 1H), 8.64 (s, 1H); ^13^C NMR (100 MHz, CDCl_3_, δ ppm): 51.51, 118.77, 121.99, 123.00, 125.79, 126.05, 126.26, 126.60, 128.27, 128.89, 130.13, 130.35, 138.90, 141.01, 146.45, 148.71, 153.79, 161.91, 168.71; GC-MS: 339 [M]^+^; Anal. calcd for C_19_H_14_ClNO_3_: Found: C, 67.18; H, 4.17; N, 4.09%.

#### 5.2.12. 3-((1Z,14E)-3-(*m*-tolylimino)-1-chloroprop-1-enyl)-2H-chromen-2-one (1l)

Light yellow crystals; Mp 212–214°C; IR (KBr) (*v*_max_/cm^−1^): 1718 (C=O of coumarin), 1627 (C=N of azomethane) cm^−1^; ^1^H NMR (400 MHz, CDCl_3_, δ ppm): 2.329 (s, 3H), 6.524 (d, 1H), 7.083–7.41 (m, Ar-H, 4H), 7.436 (t, 1H, *J *= 7.6 Hz), 7.462 (d, 1H, *J *= 8.1 Hz), 7.770 (t, 1H, *J *= 8.0 Hz), 8.046 (d, 1H), 8.28 (d, 1H), 8.714 (s, 1H); ^13^C NMR (100 MHz, CDCl_3_, δ ppm): 22.16, 119.22, 122.77, 125.64, 125.97, 126.65, 126.75, 128.85, 128.93, 129.44, 129.80, 130.60, 131.20, 131.70, 134.23, 139.76, 149.27, 154.41, 169.16; GC-MS: 323 [M]^+^; Anal. calcd for C_19_H_14_ClNO_2_: Found: C, 70.55; H, 4.41; N, 4.29%.

## Supplementary Material

Spectral Data

## Data accessibility

The datasets supporting this article have been uploaded as part of the electronic supplementary material.
