# Supplementary material for: Functionalization of 3-chloroformylcoumarin to coumarin Schiff bases using reusable catalyst: an approach to molecular docking and biological studies
Source: R Soc Open Sci. 2018 May 2;5(5):172416. doi: 10.1098/rsos.172416 (PMC5990764; doi:10.1098/rsos.172416)
Supplement: Spectral Data [file rsos172416supp1.docx]

Functionalization of 3-Chloroformylcoumarin to coumarin-Schiff Bases using Reusable Catalyst: an approach to molecular docking and Biological studies

Suresh S. Kumbar,^[a]^ Kallappa M. Hosamani,^*[a]^ Gangadhar C. Gouripur,^[b]^ and Shrinivas D. Joshi^[c]^

^[a]^ Department of Studies in Chemistry, Karnatak University, Dharwad-580003, Karnataka, INDIA.

^[b]^ P. G. Department of Biotechnology and Microbiology, Karnatak University, Dharwad-580003, Karnataka, INDIA.

^[c]^ Novel Drug Design and Discovery Laboratory, Department of Pharmaceutical Chemistry, S.E.T.’s College of Pharmacy, Sangolli Rayanna Nagar, Dharwad 580002, Karnataka, INDIA.

*Corresponding author e-mail: dr_hosamani@yahoo.com (Dr. K. M. Hosamani)

Supplementary Data of (**1a-1l**) compounds

- IR
- ^1^H NMR
- ^13^C NMR
- GC-MS & ESI spectrum


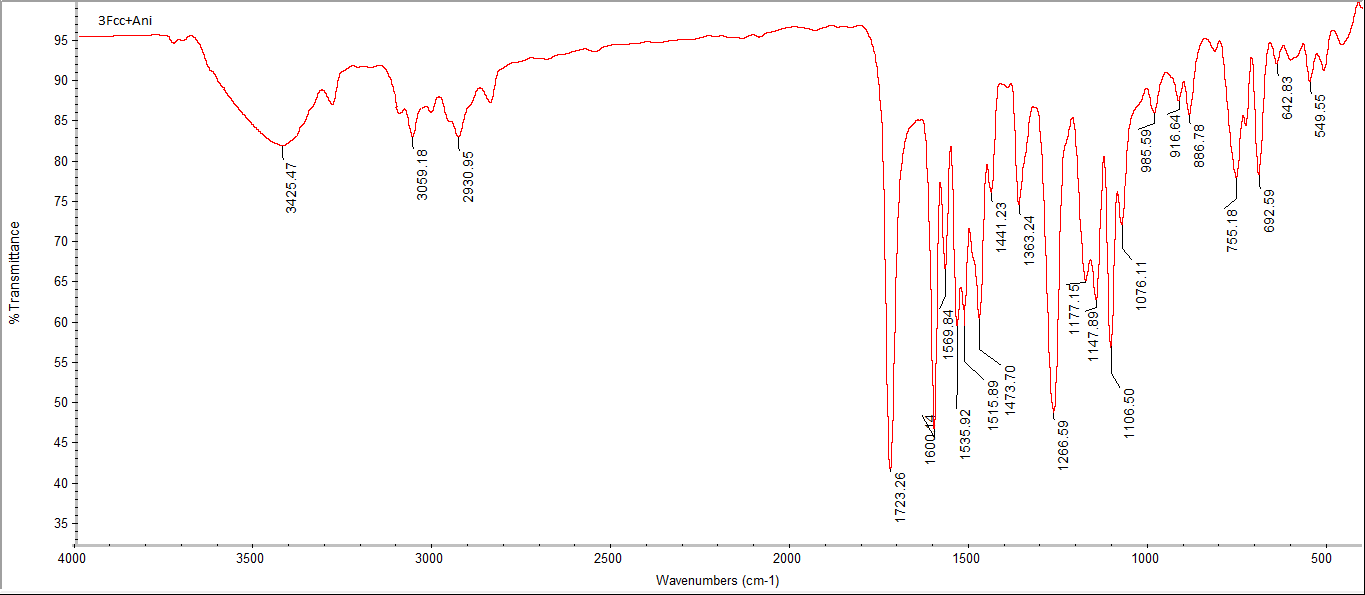

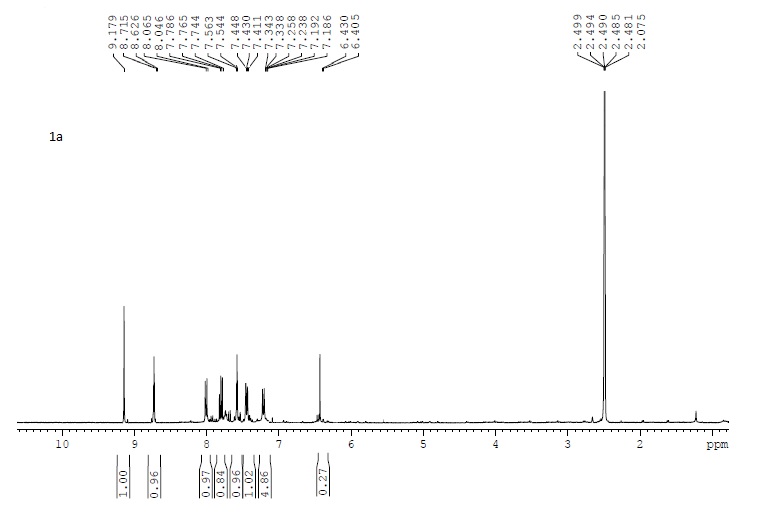
IR Spectra of **1a**

^1^H NMR Spectra of **1a**

^
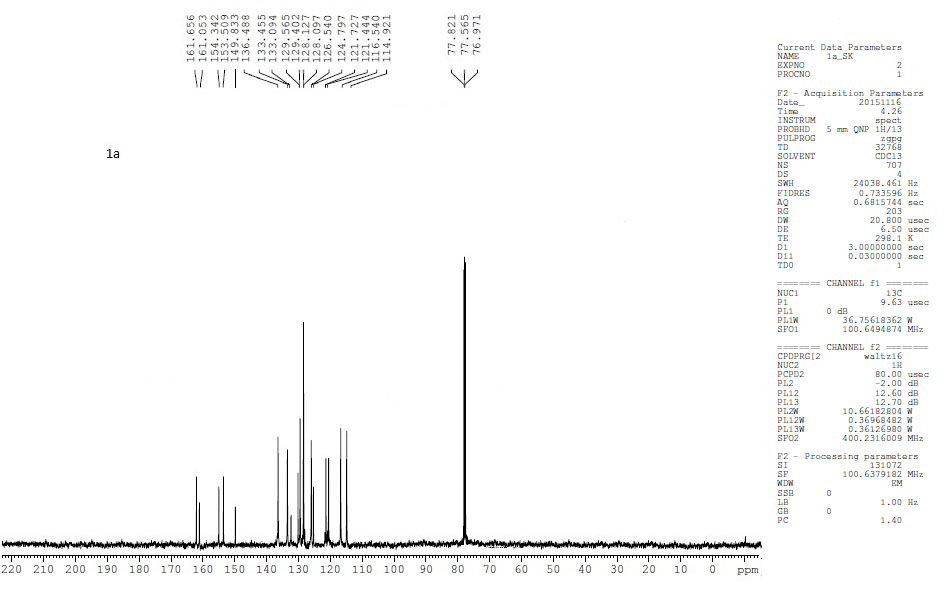
13^C NMR Spectra of **1a**


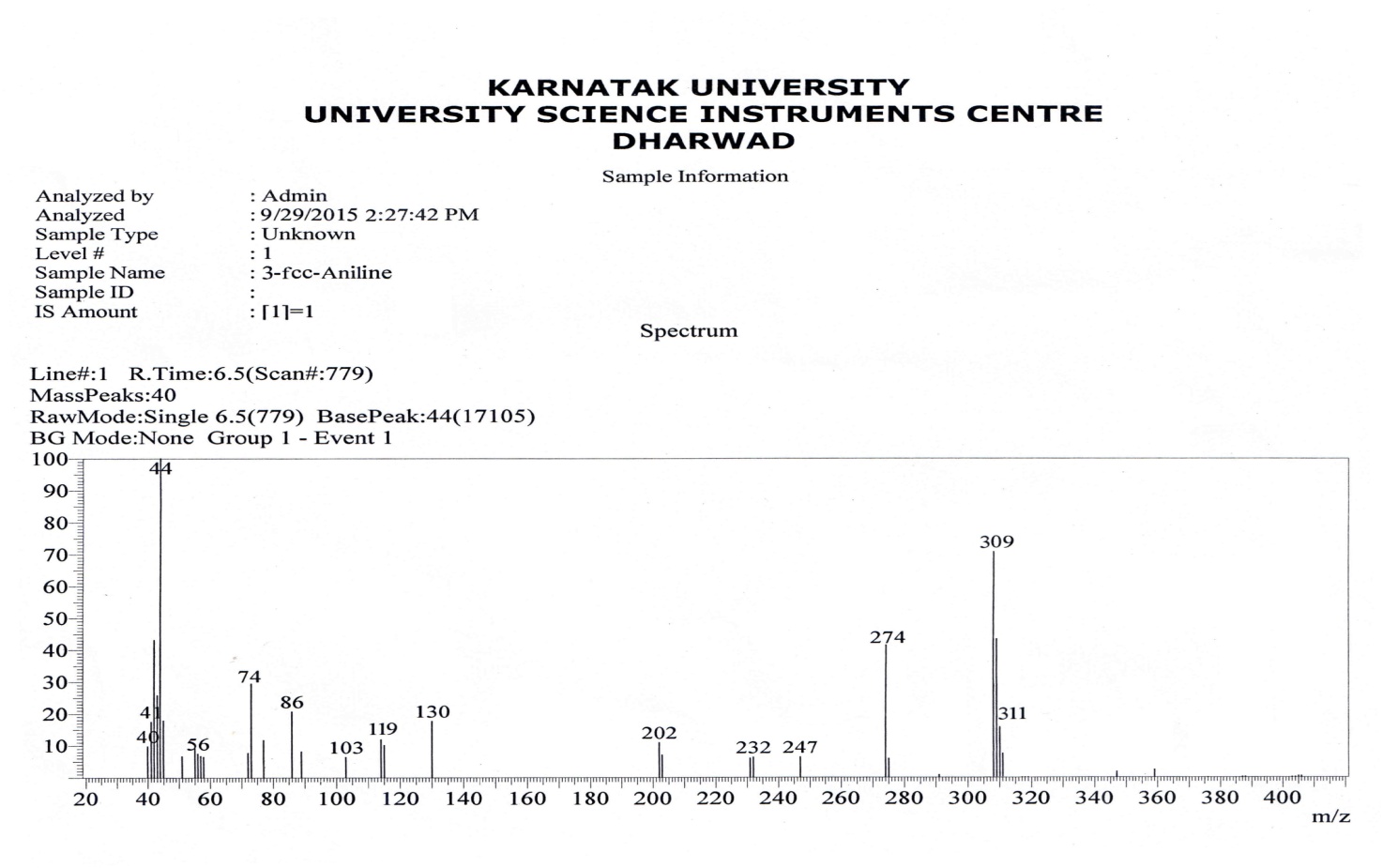


Mass Spectra of **1a**


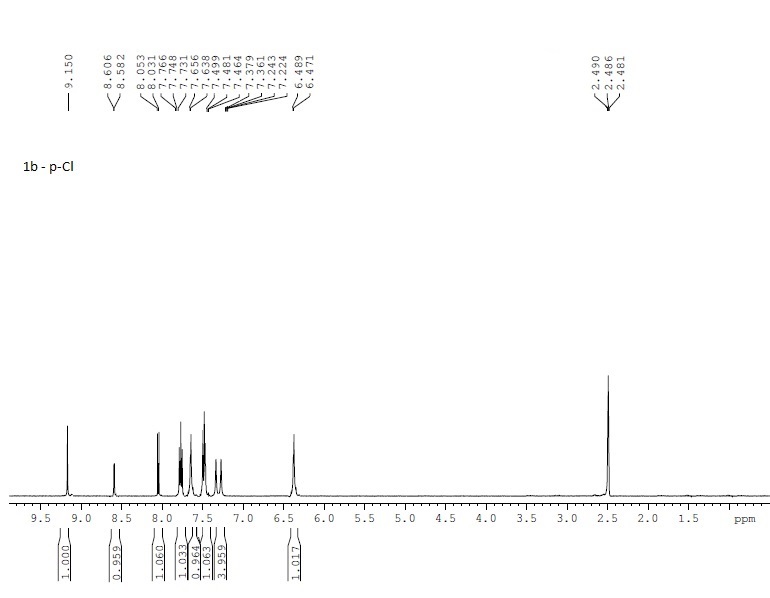
IR Spectra of **1
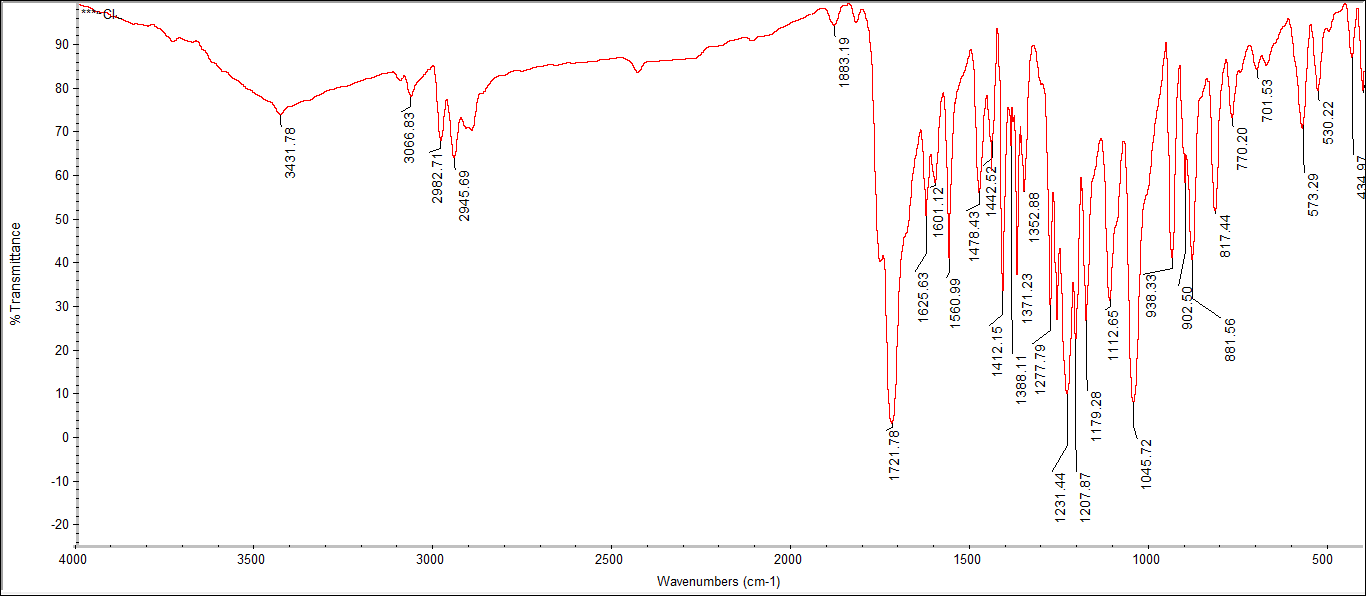
b**

^1^H NMR Spectra of **1b**


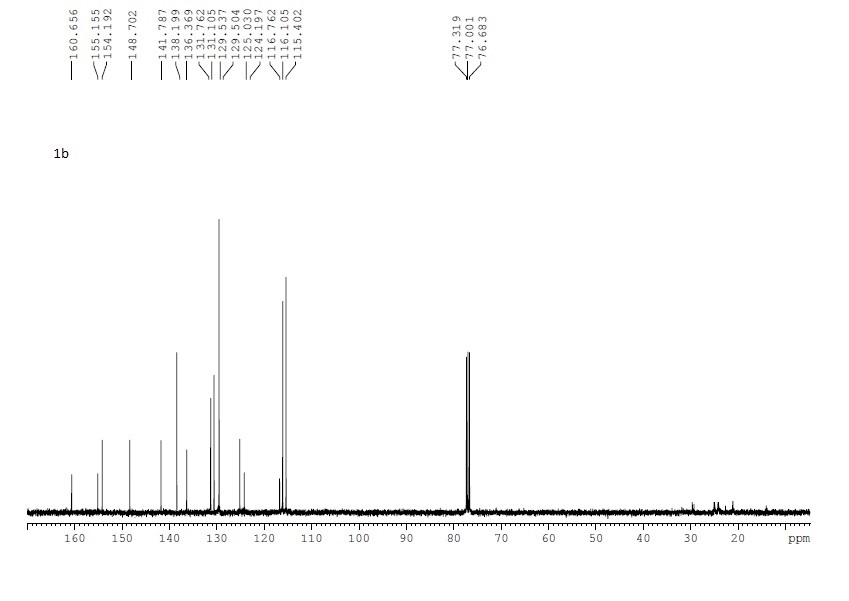


^13^C NMR Spectra of **1b**


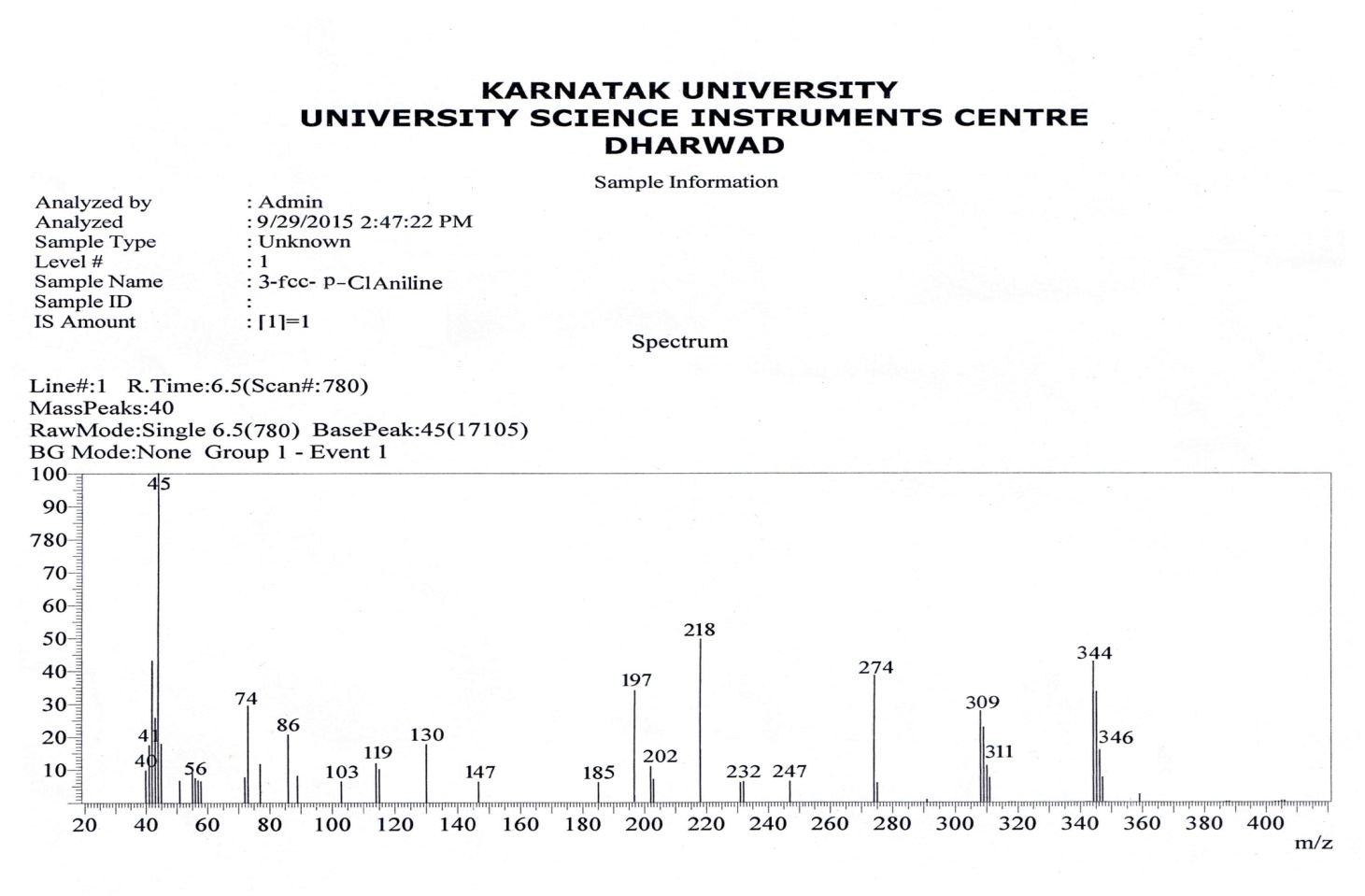


Mass Spectra of **1b**


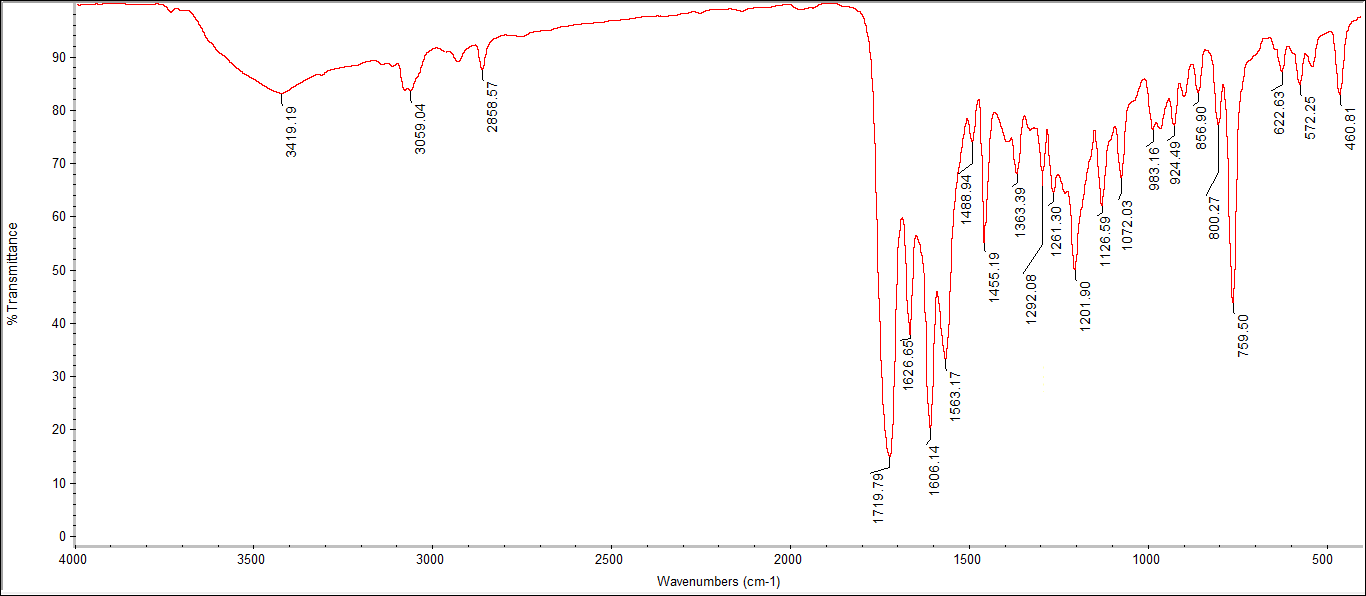
IR Spectra of **1c**

^
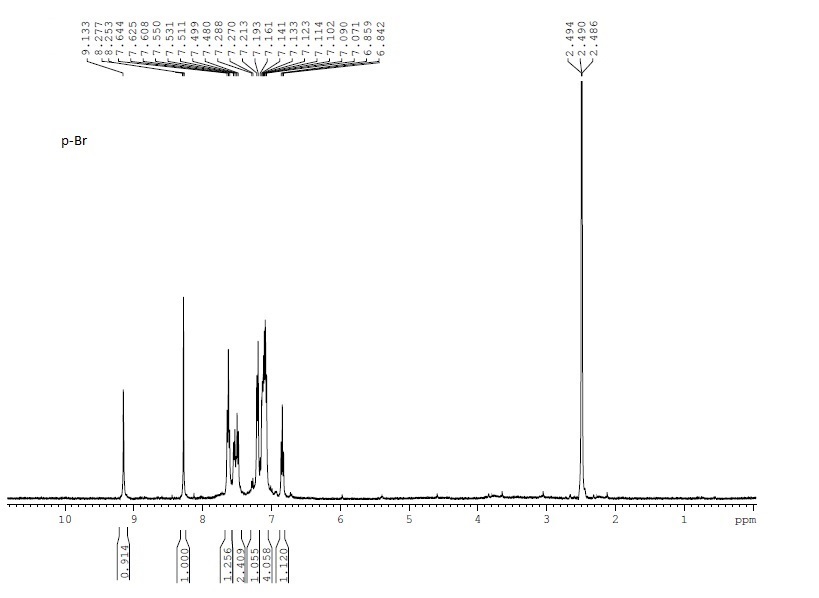
^

^1^H NMR Spectra of **1c**


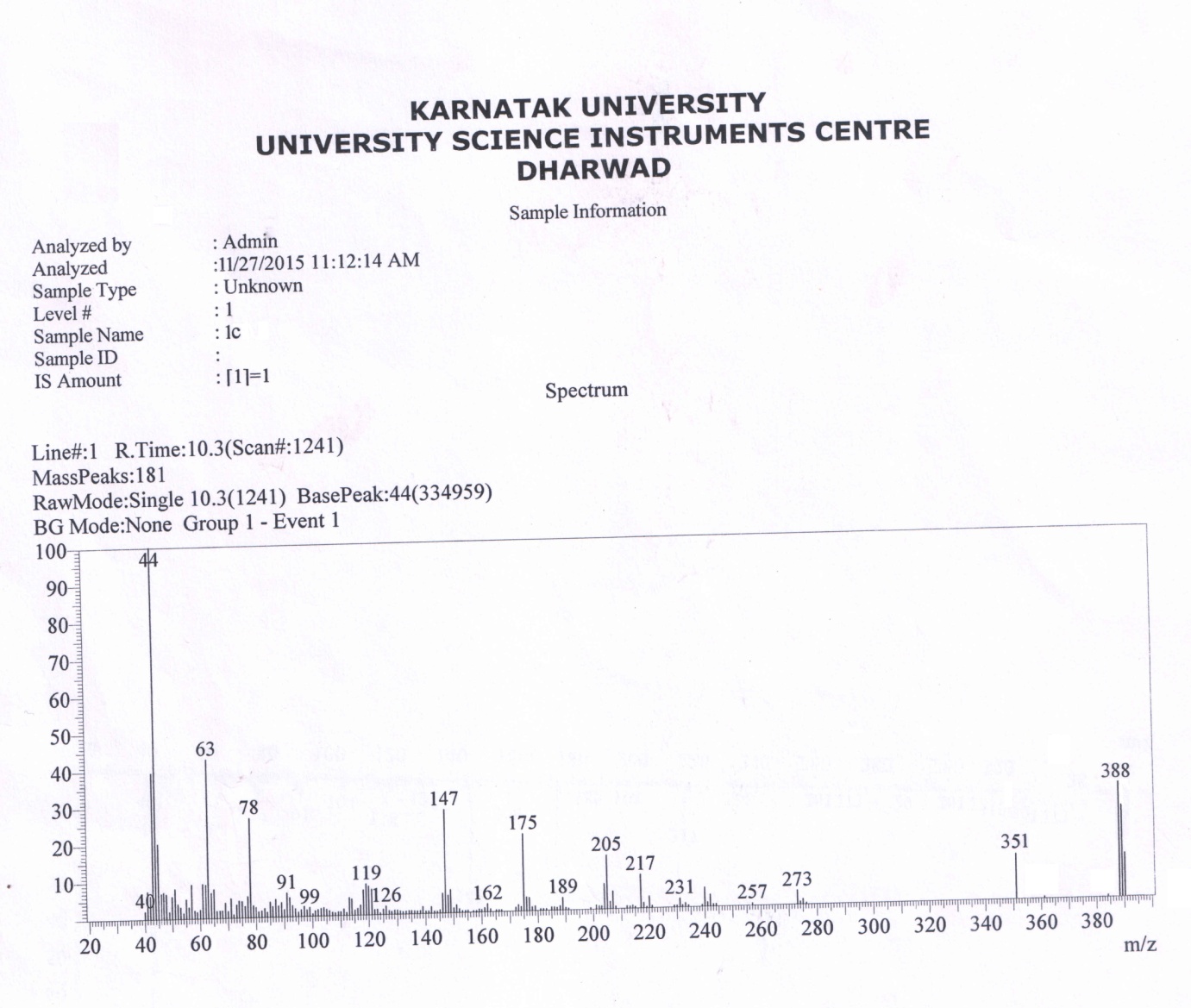

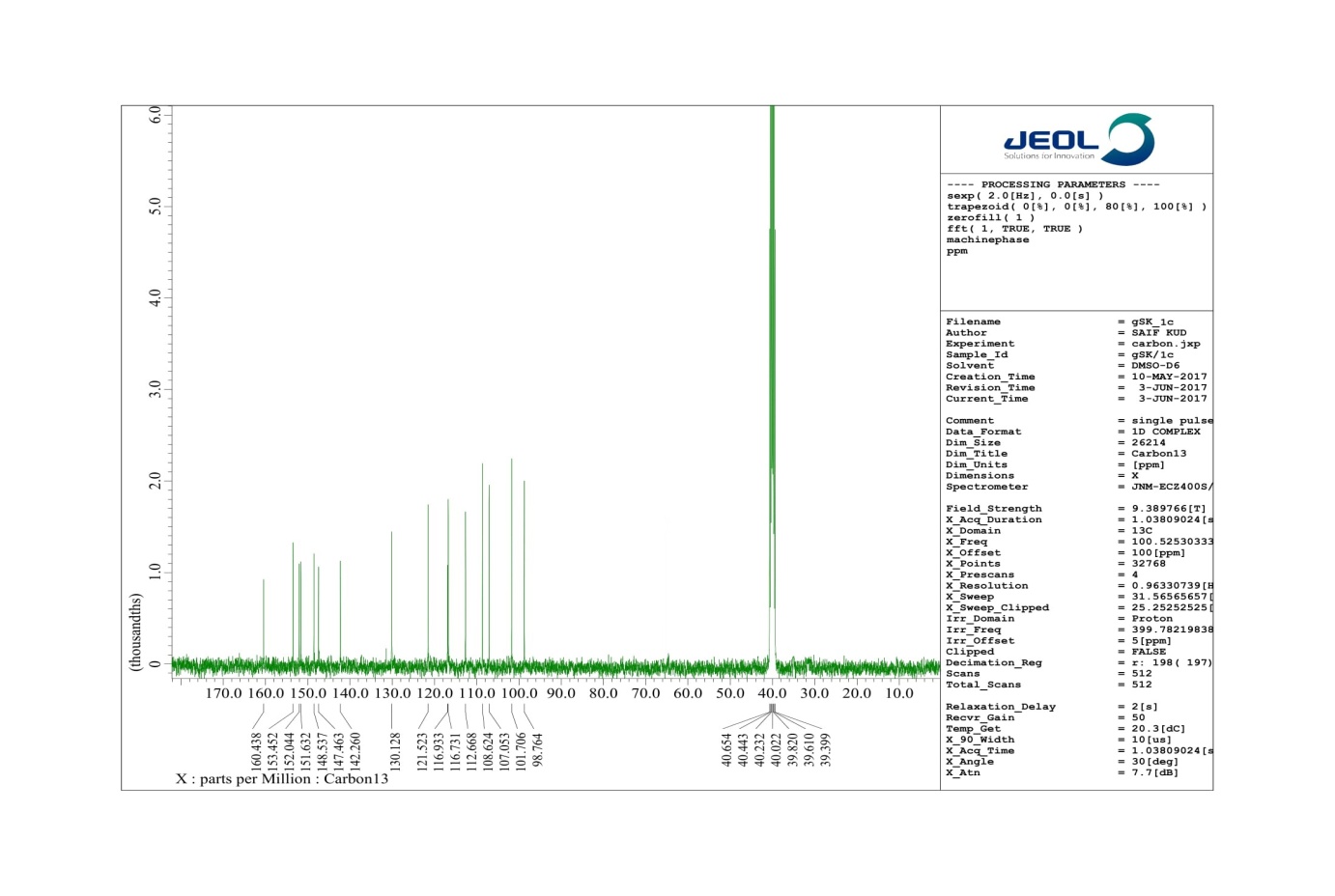
^13^C NMR Spectra of **1c**

Mass Spectra of **1c**

IR Spectra of **1
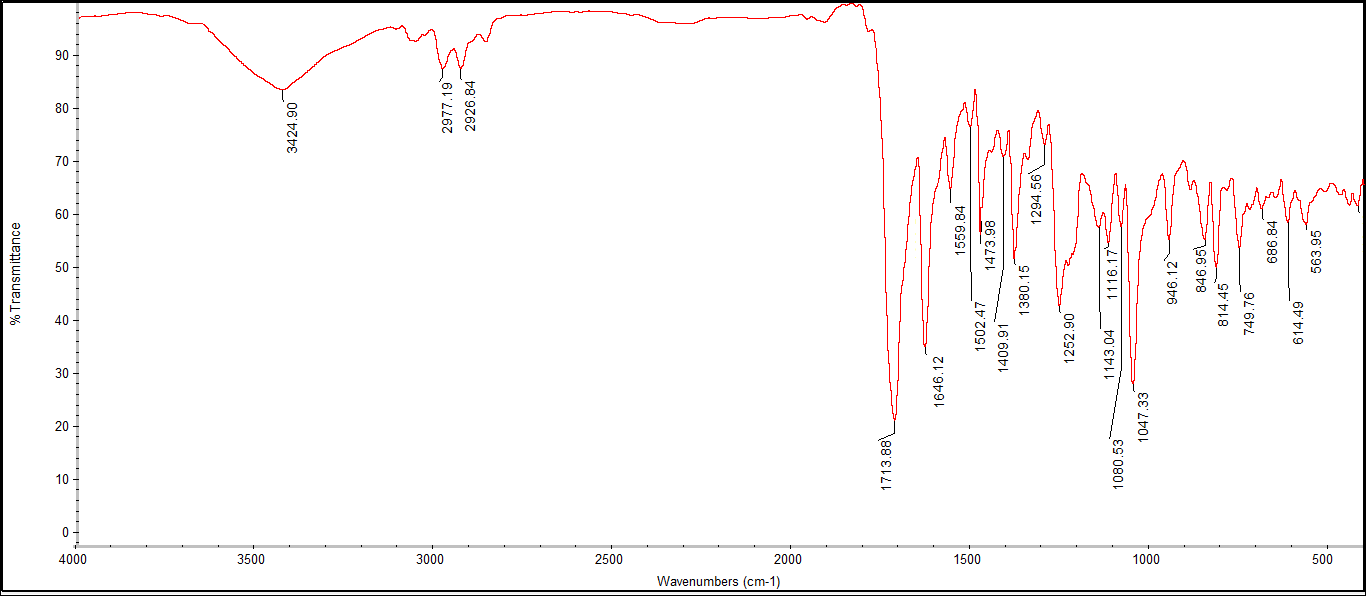
d**


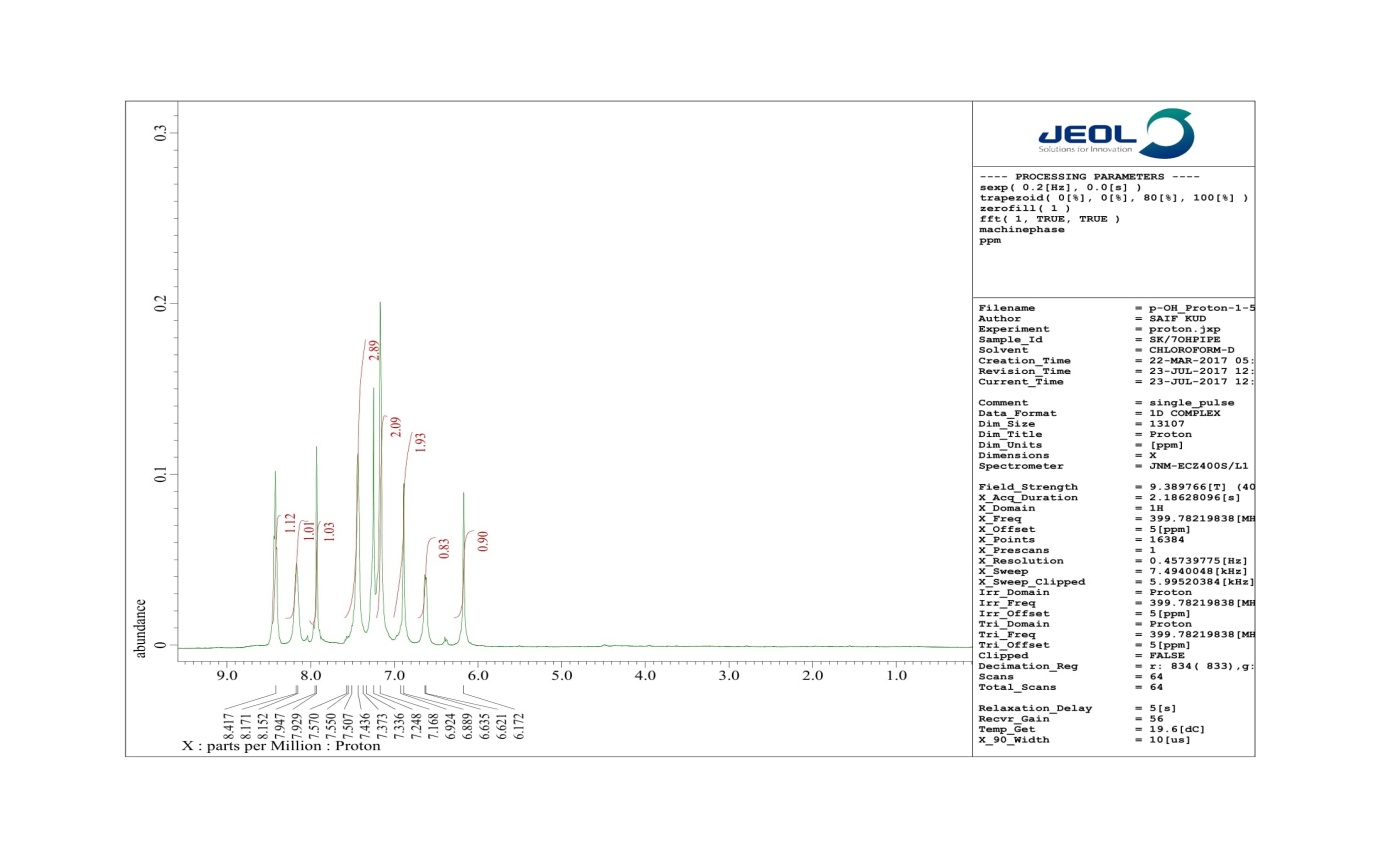


^1^H NMR Spectra of **1d**

^
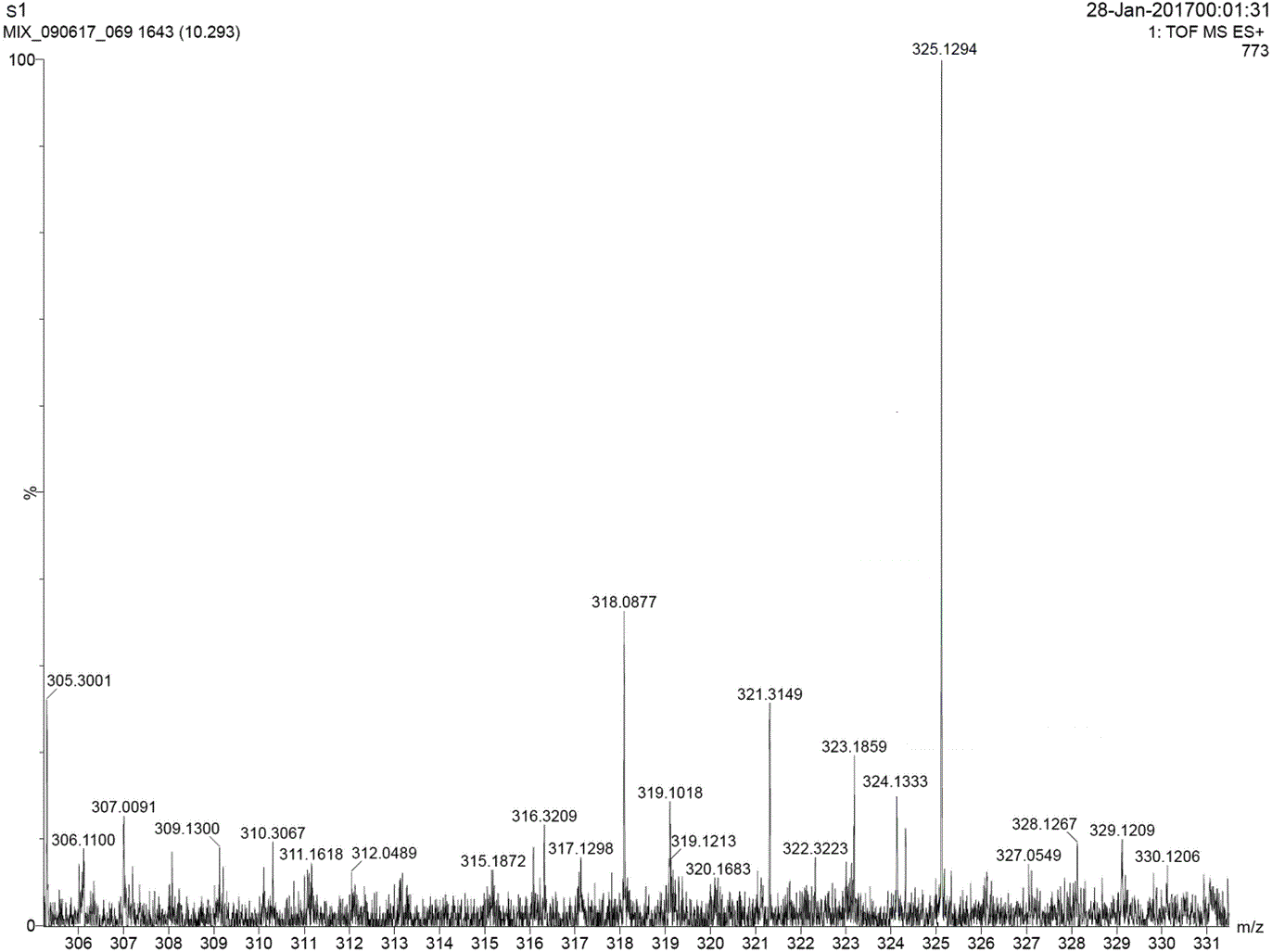
13^C NMR Spectra of **1
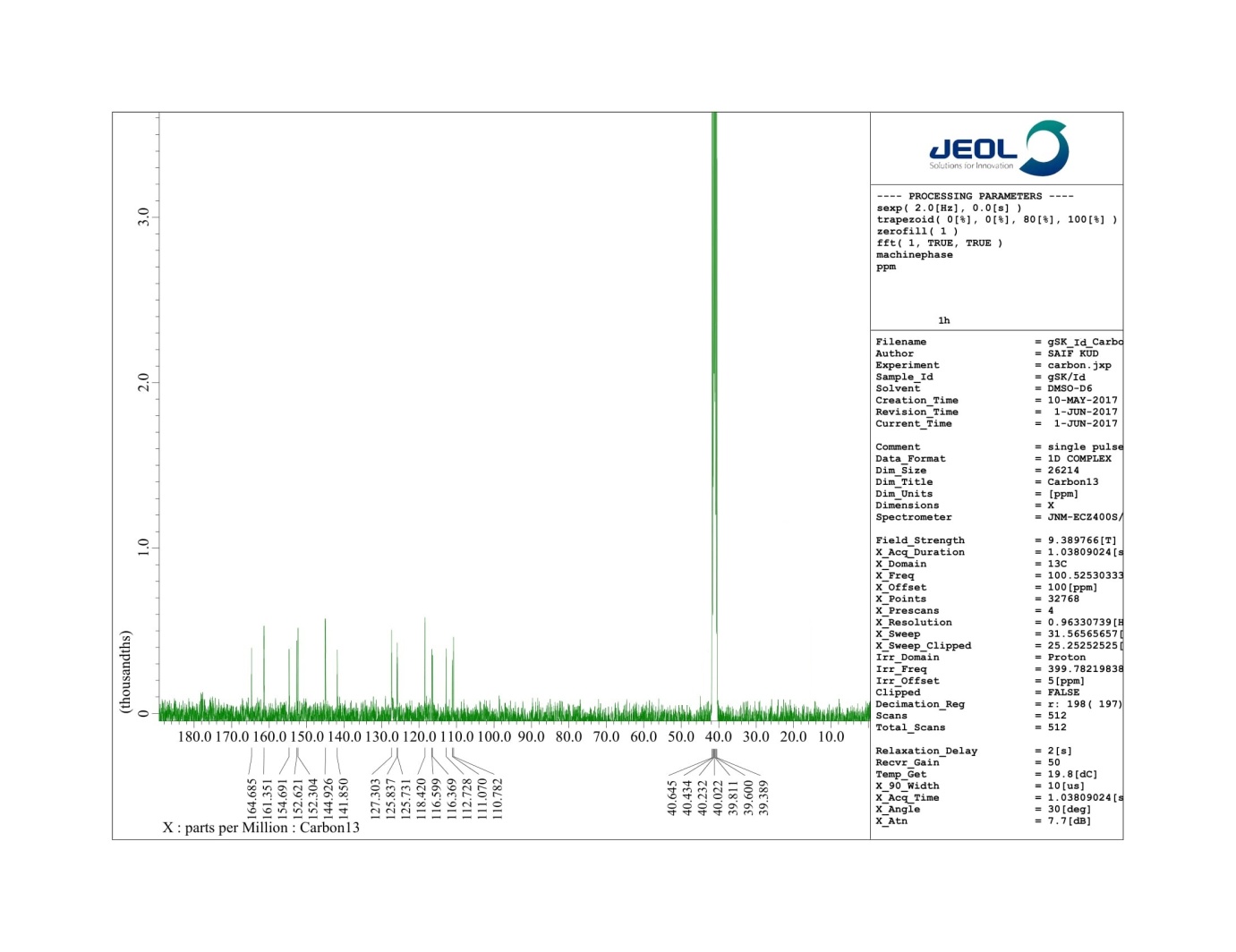
d**

ESI spectra of **1d**


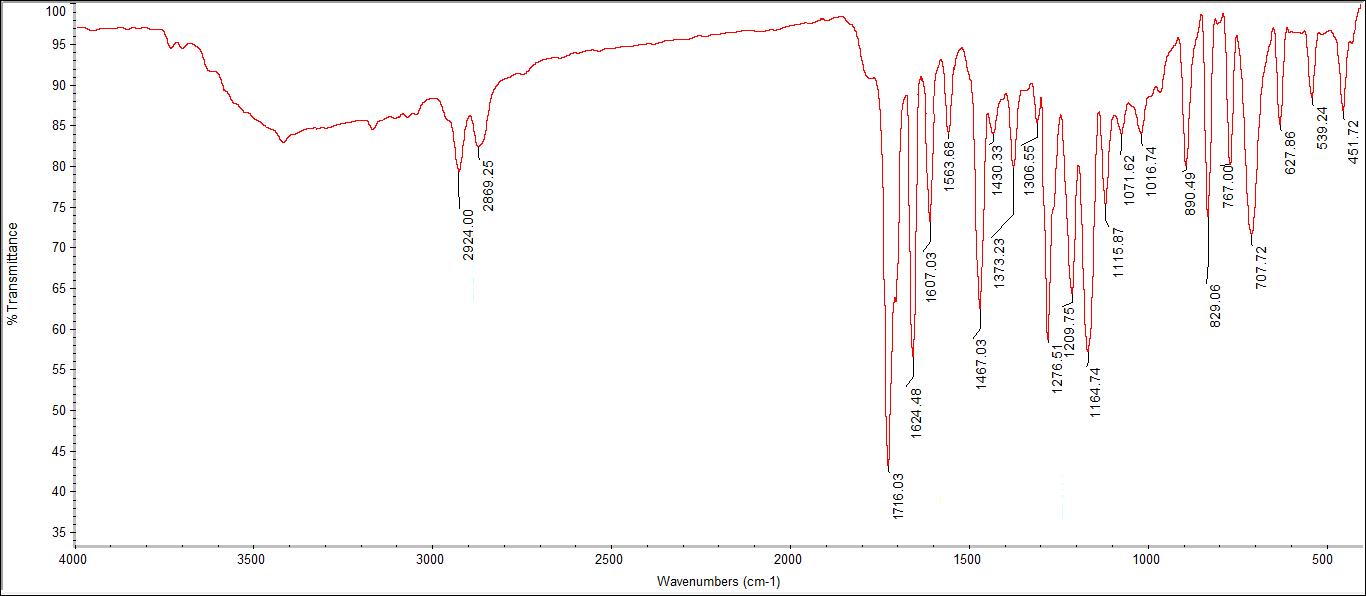
IR Spectra of **1e**

^
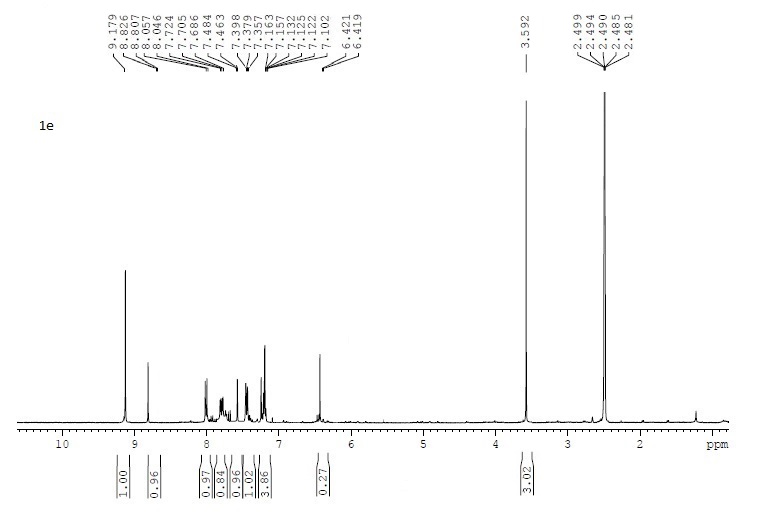
^

^1^H NMR Spectra of **1e**

^
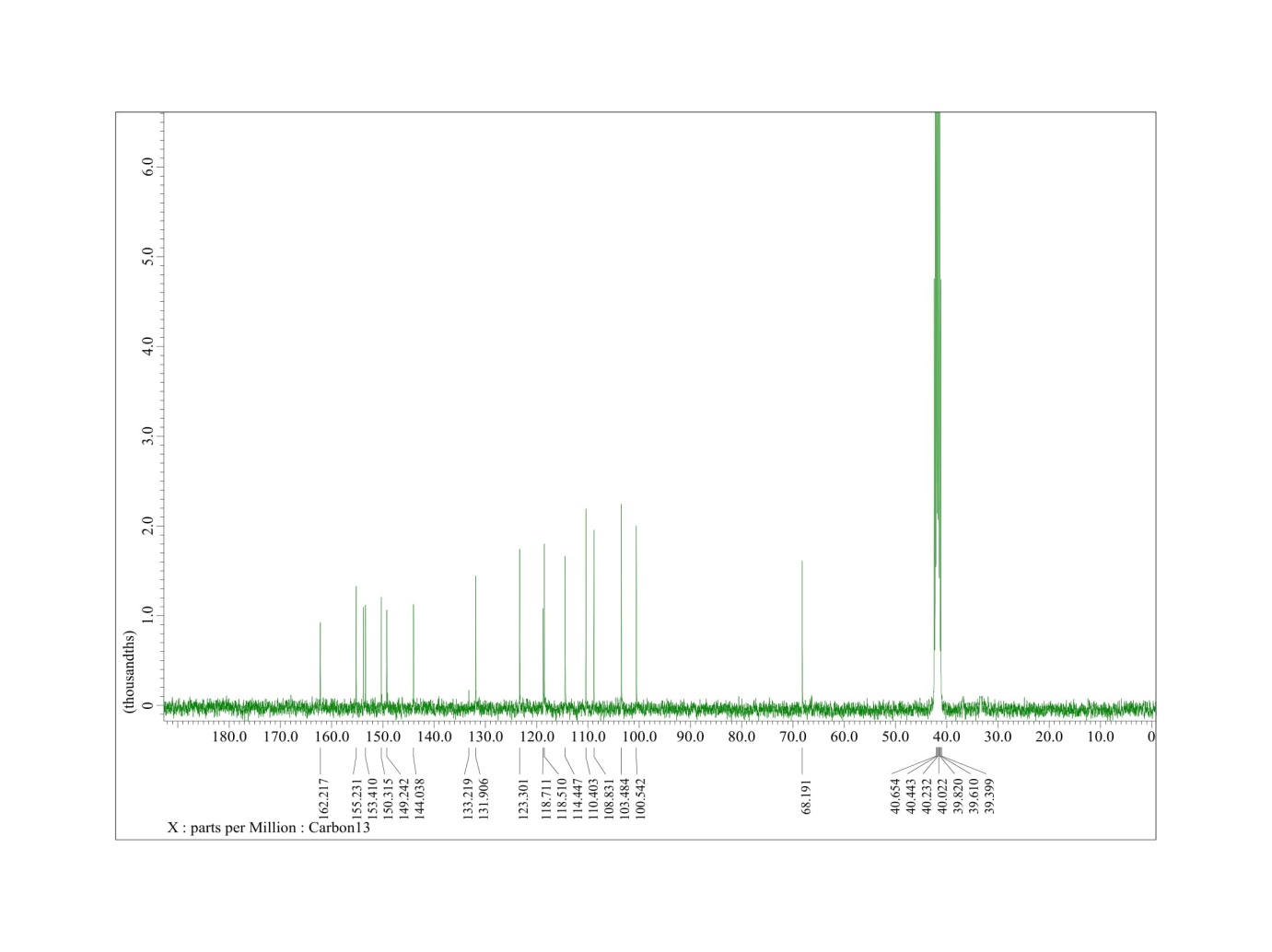
^

^13^C NMR Spectra of **1e**


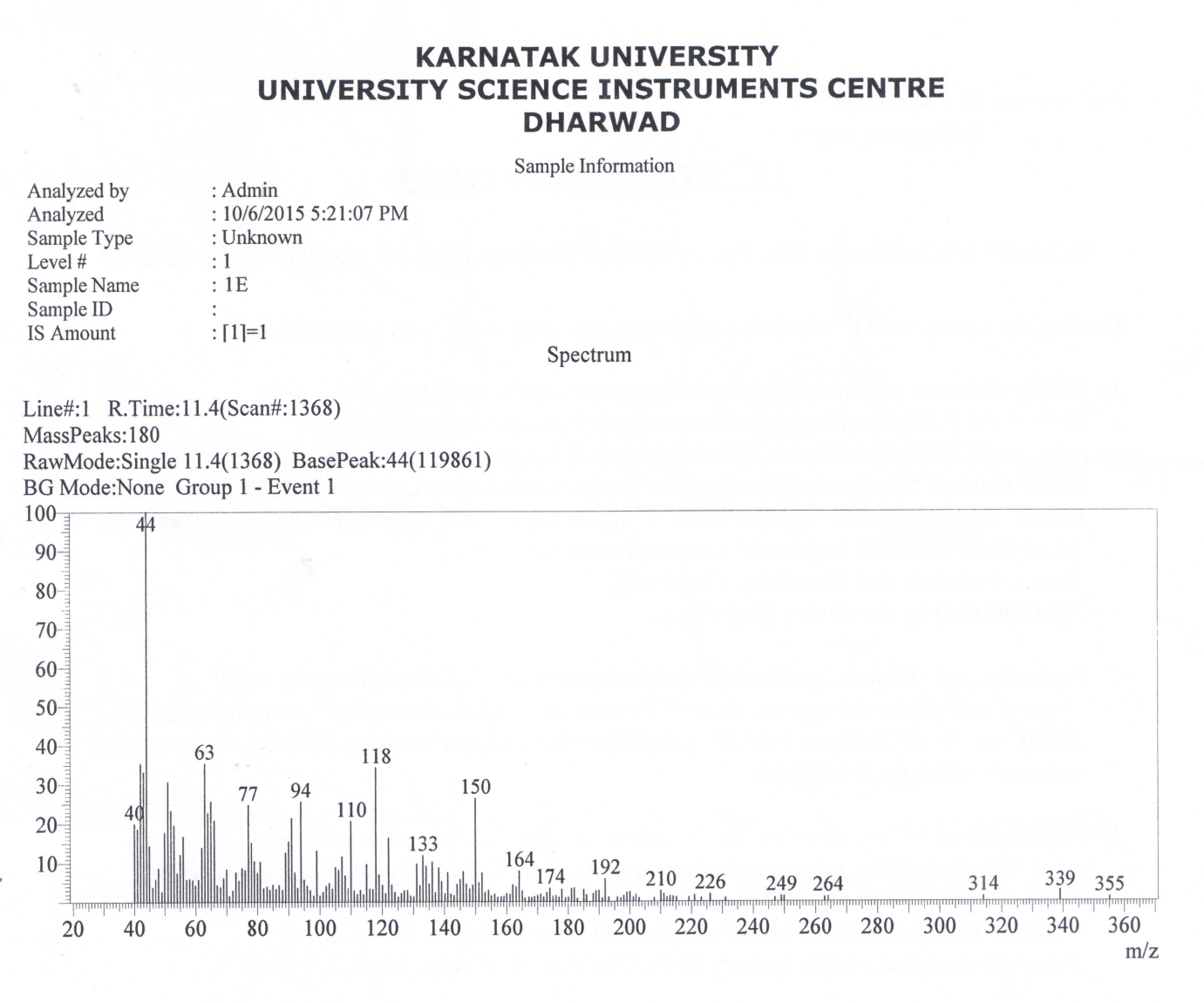


Mass Spectra of **1e**


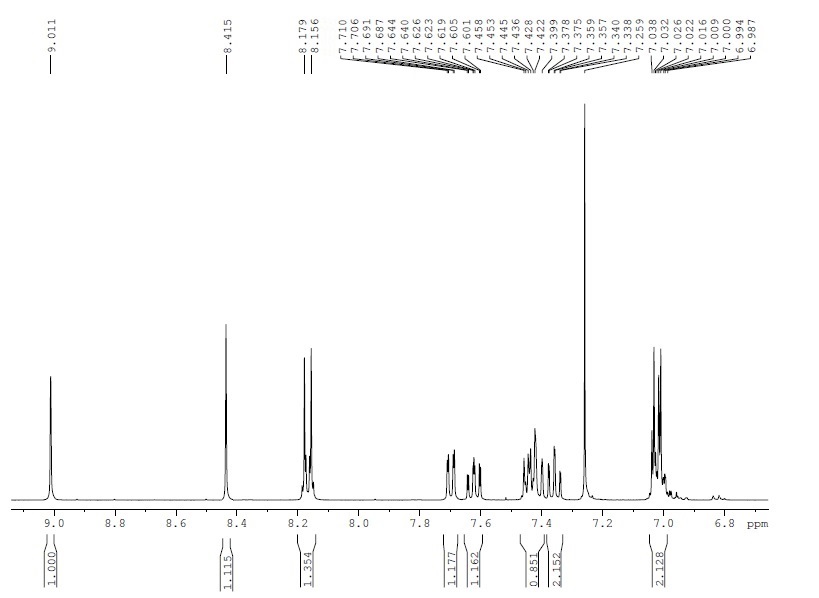
IR Spectra of **1
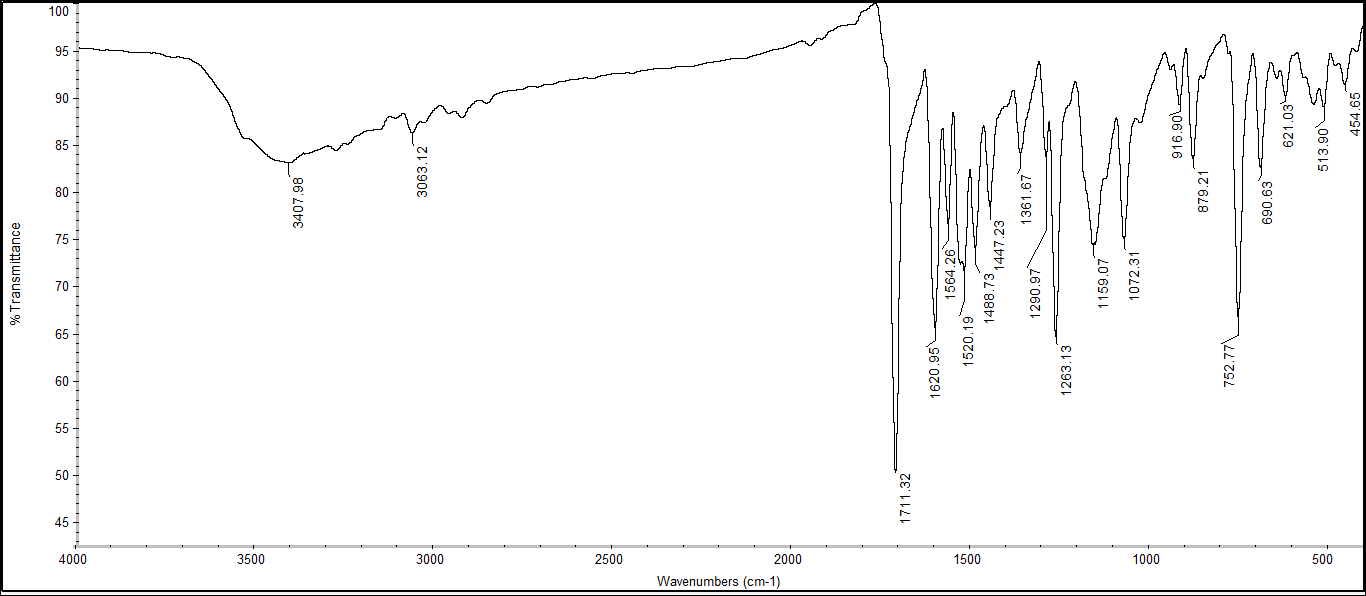
f**

^1^H NMR Spectra of **1f**


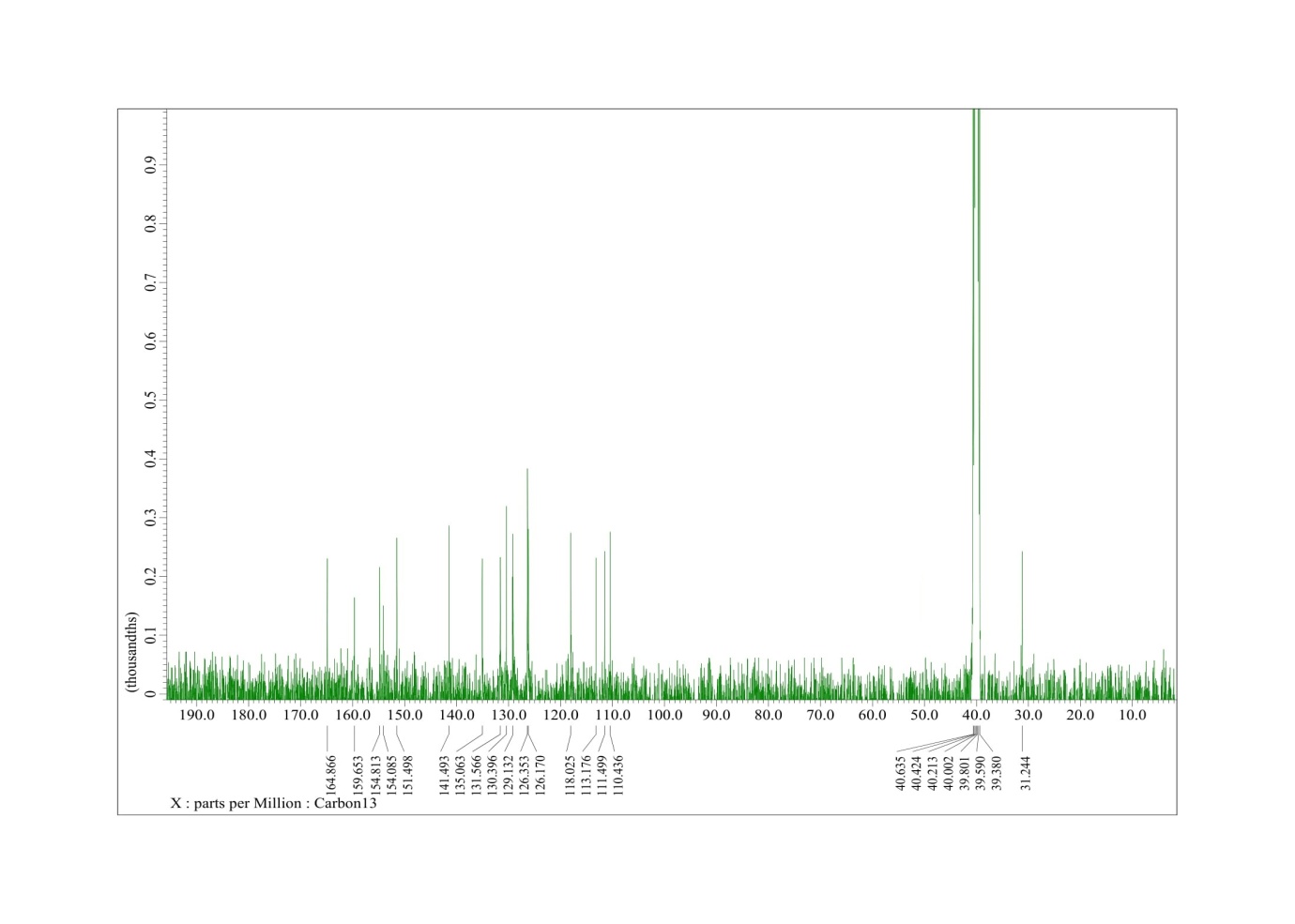


^13^C NMR Spectra of **1f**


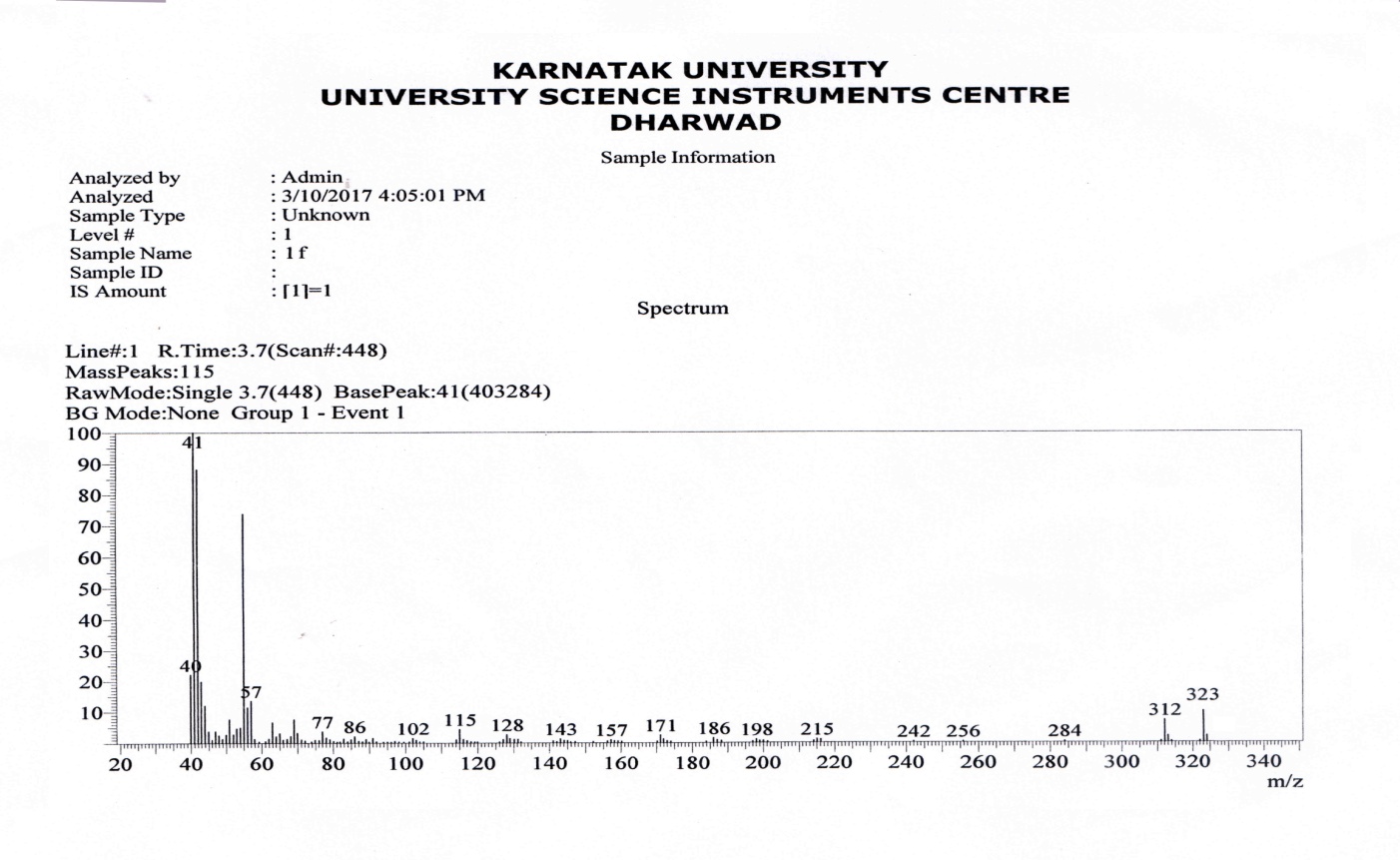


Mass Spectra of **1f**

IR Spectra of **1
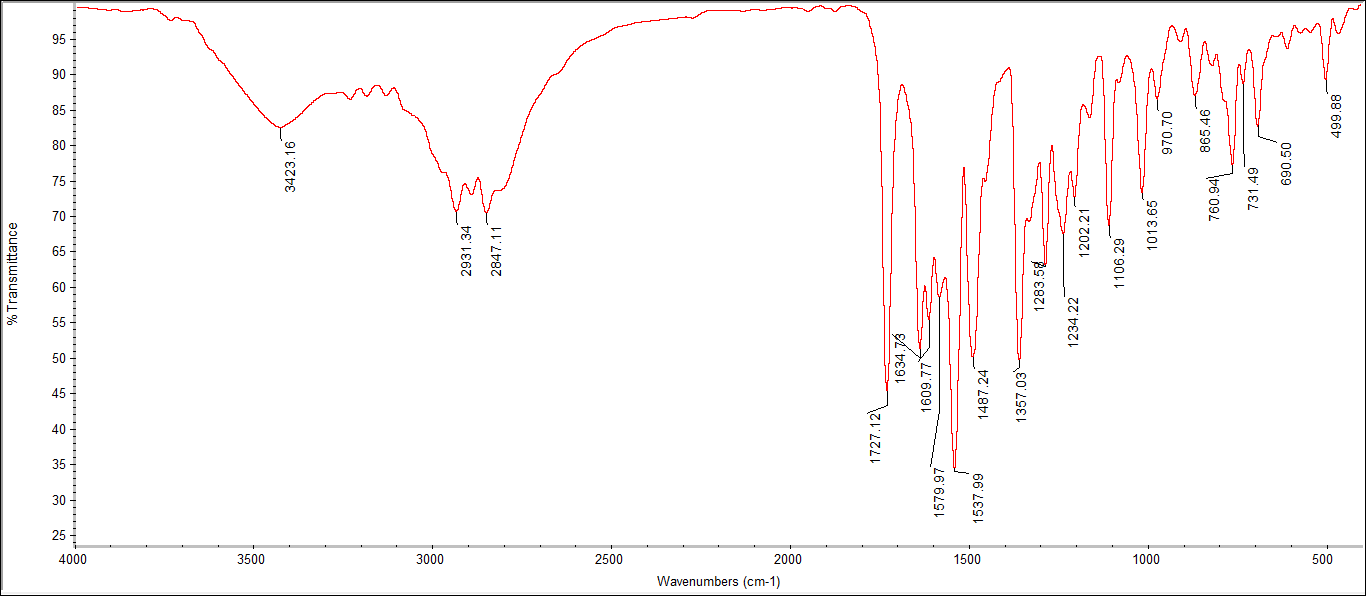
g**

^1^H NMR Spectra of **1
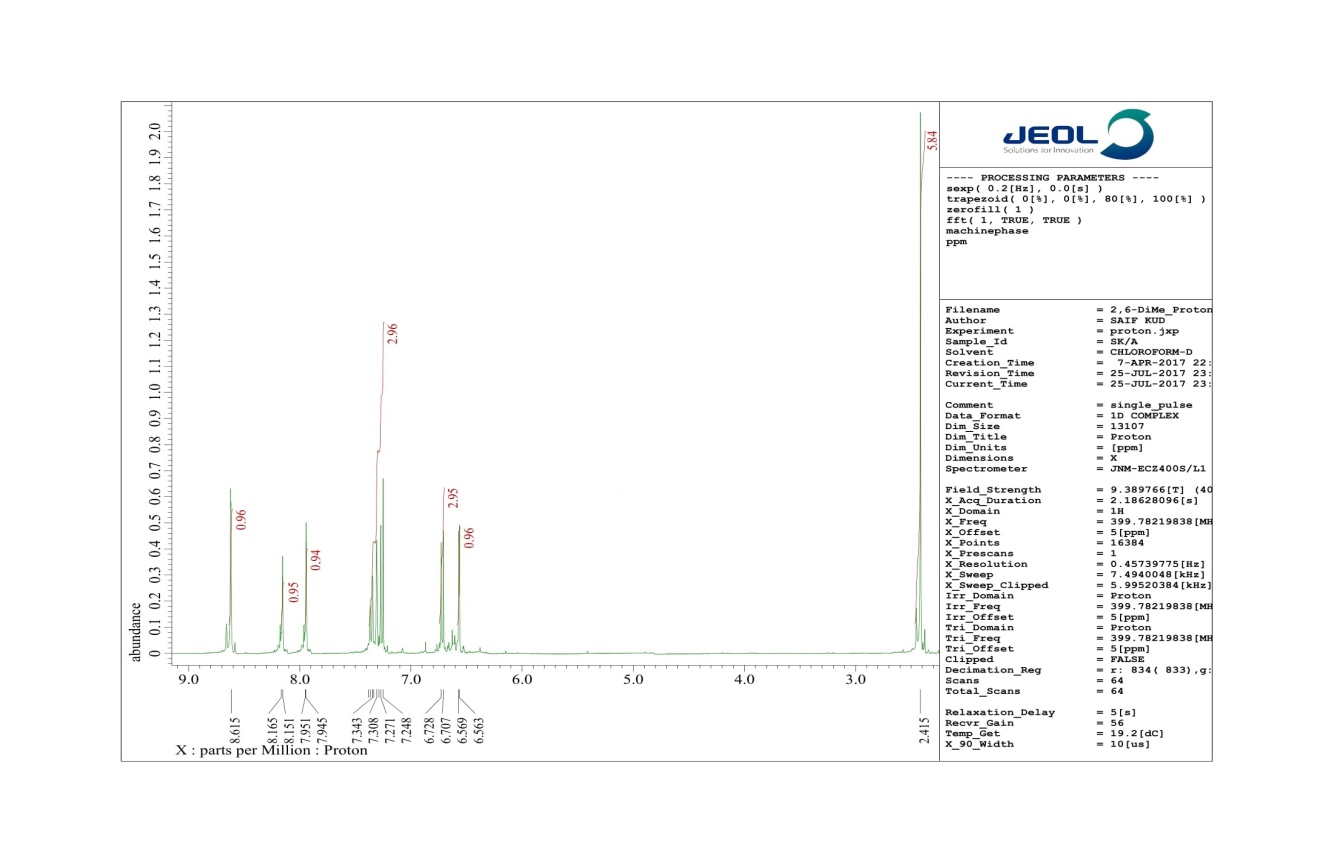
g**

^13^C NMR Spectra of **1
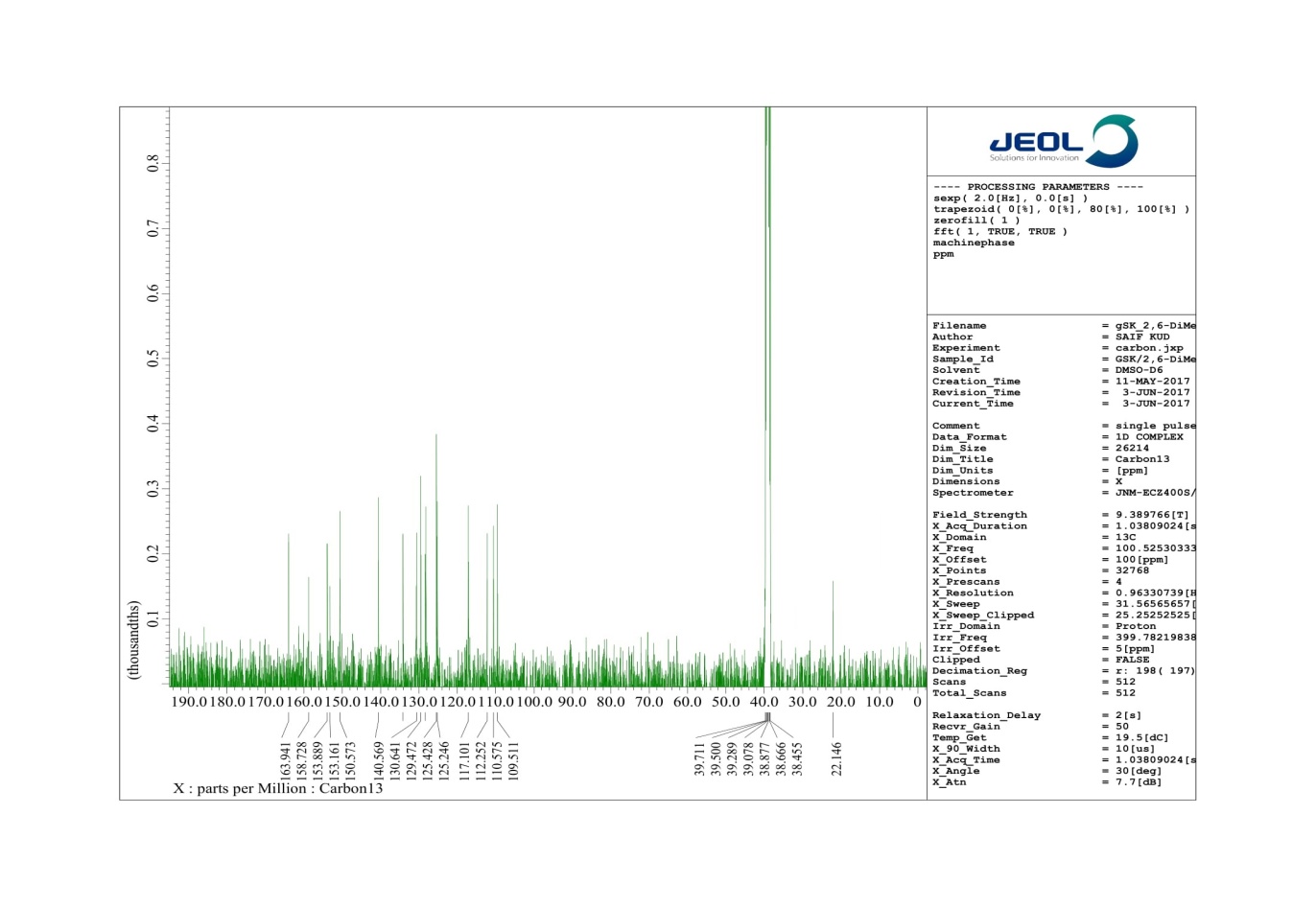
g**


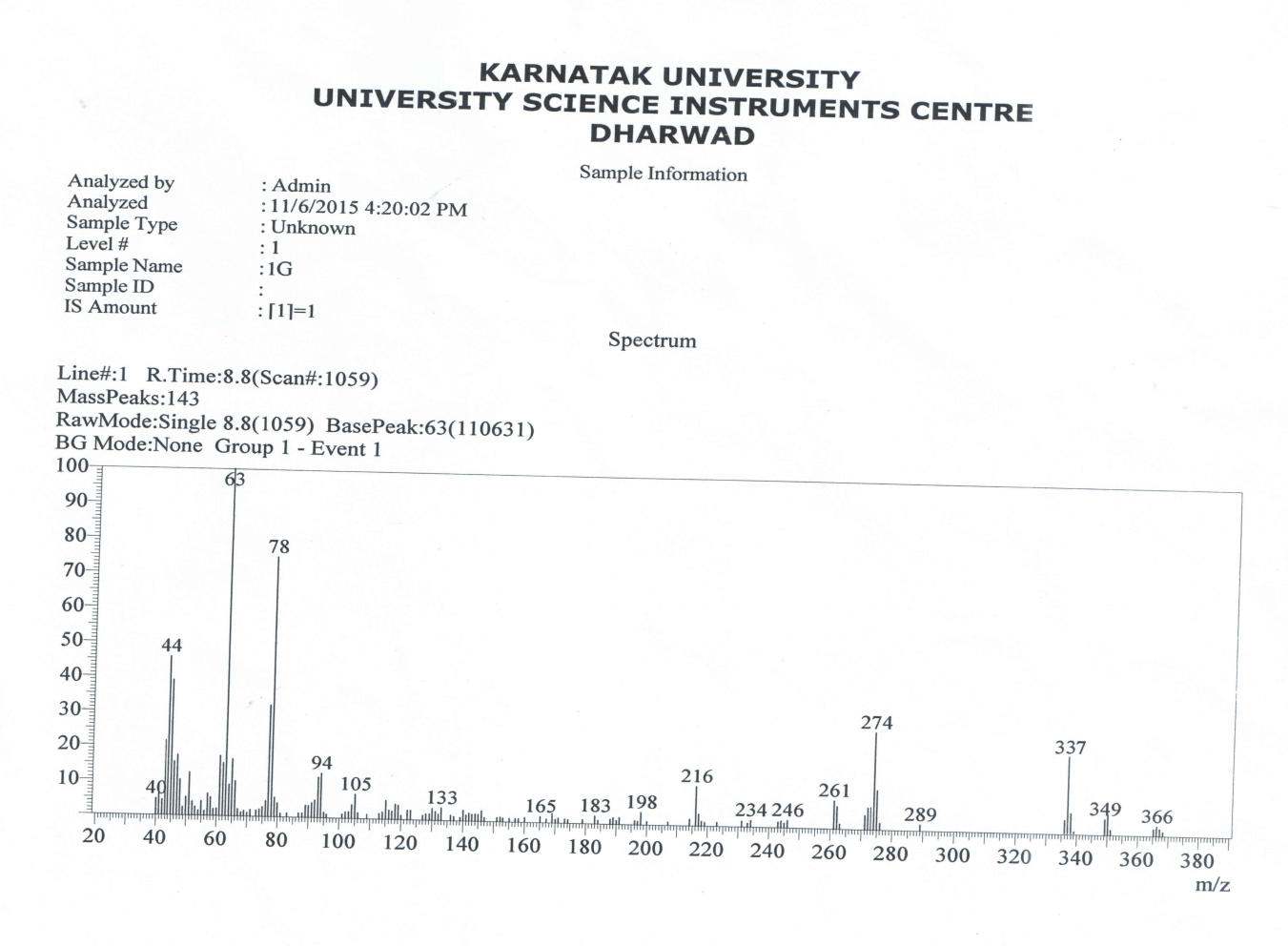


Mass Spectra of **1g**


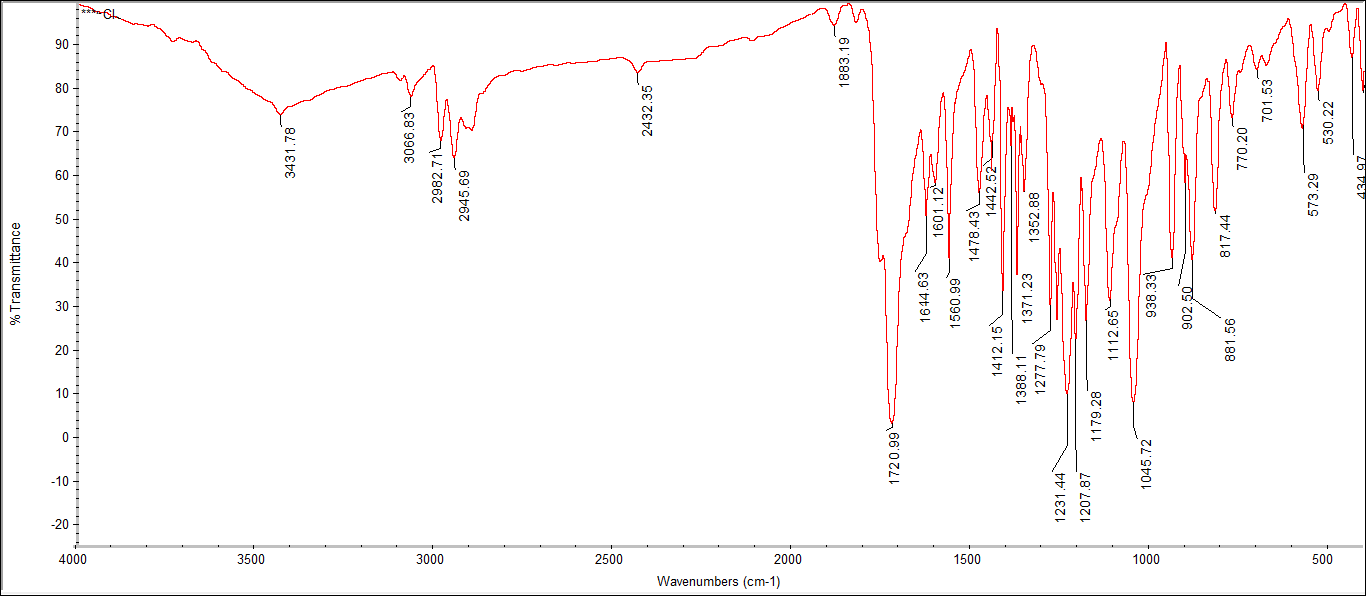
 IR Spectra of **1h**


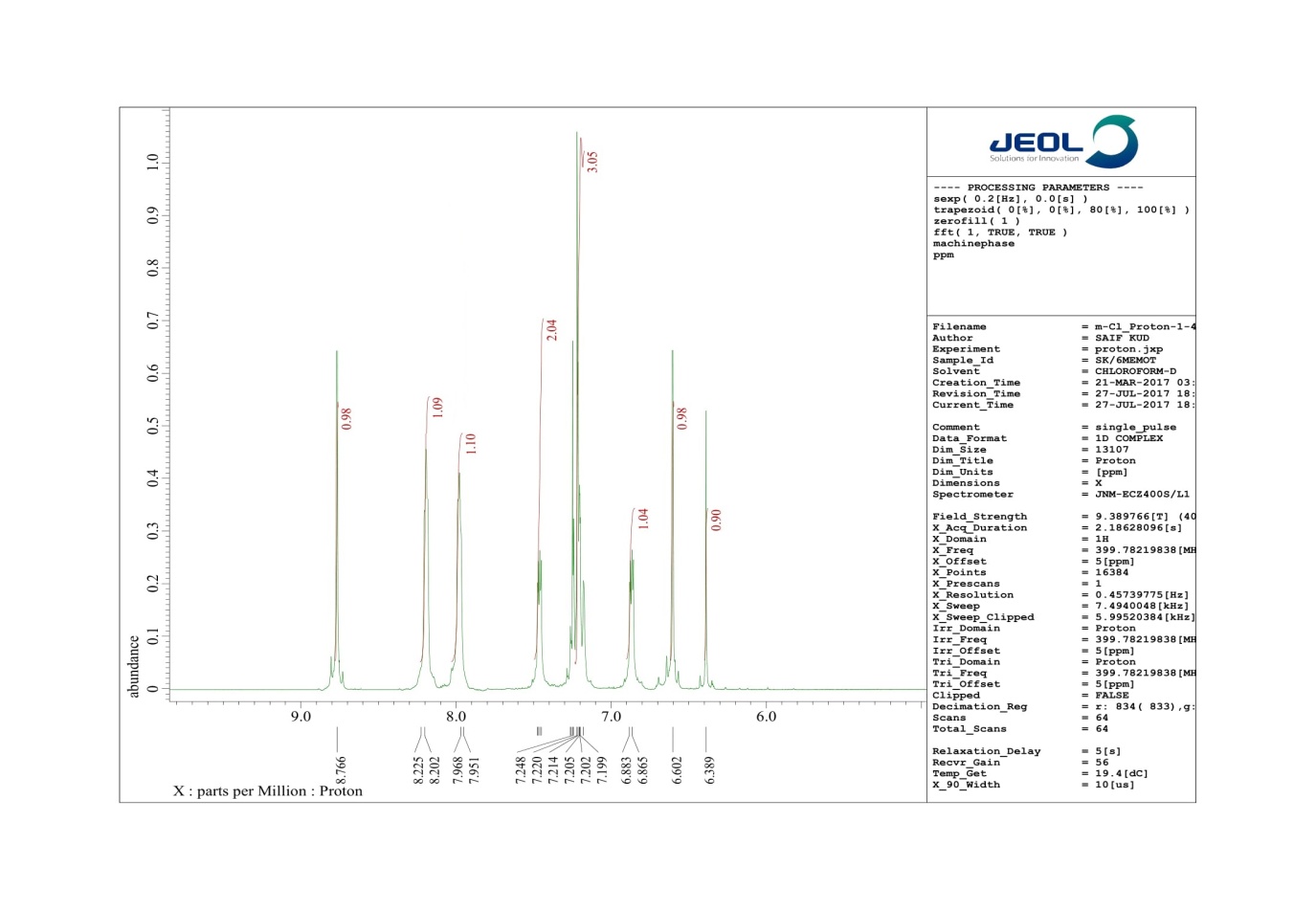


^1^H NMR Spectra of **1h**

^13^C NMR Spectra of **1
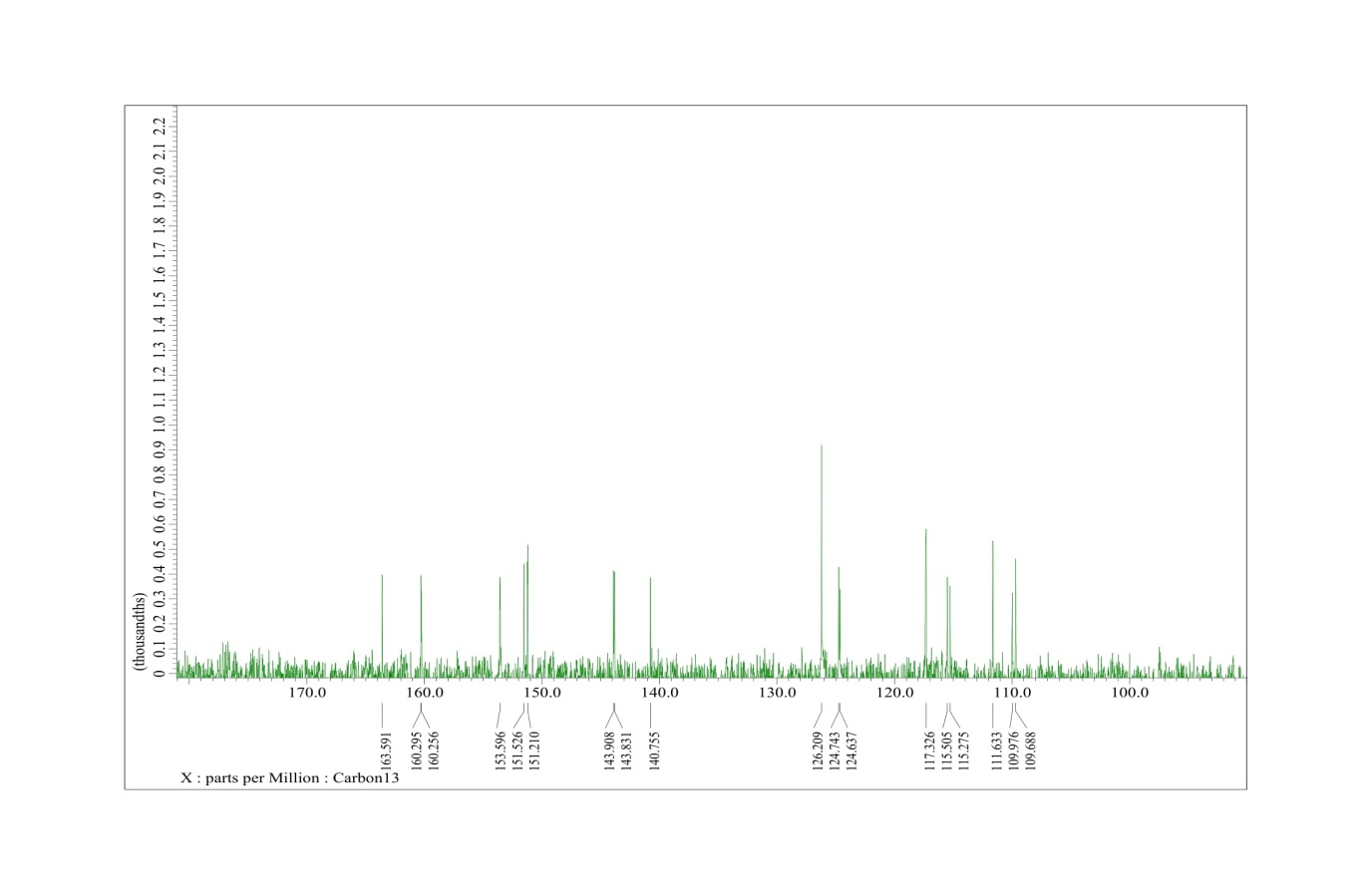
h**


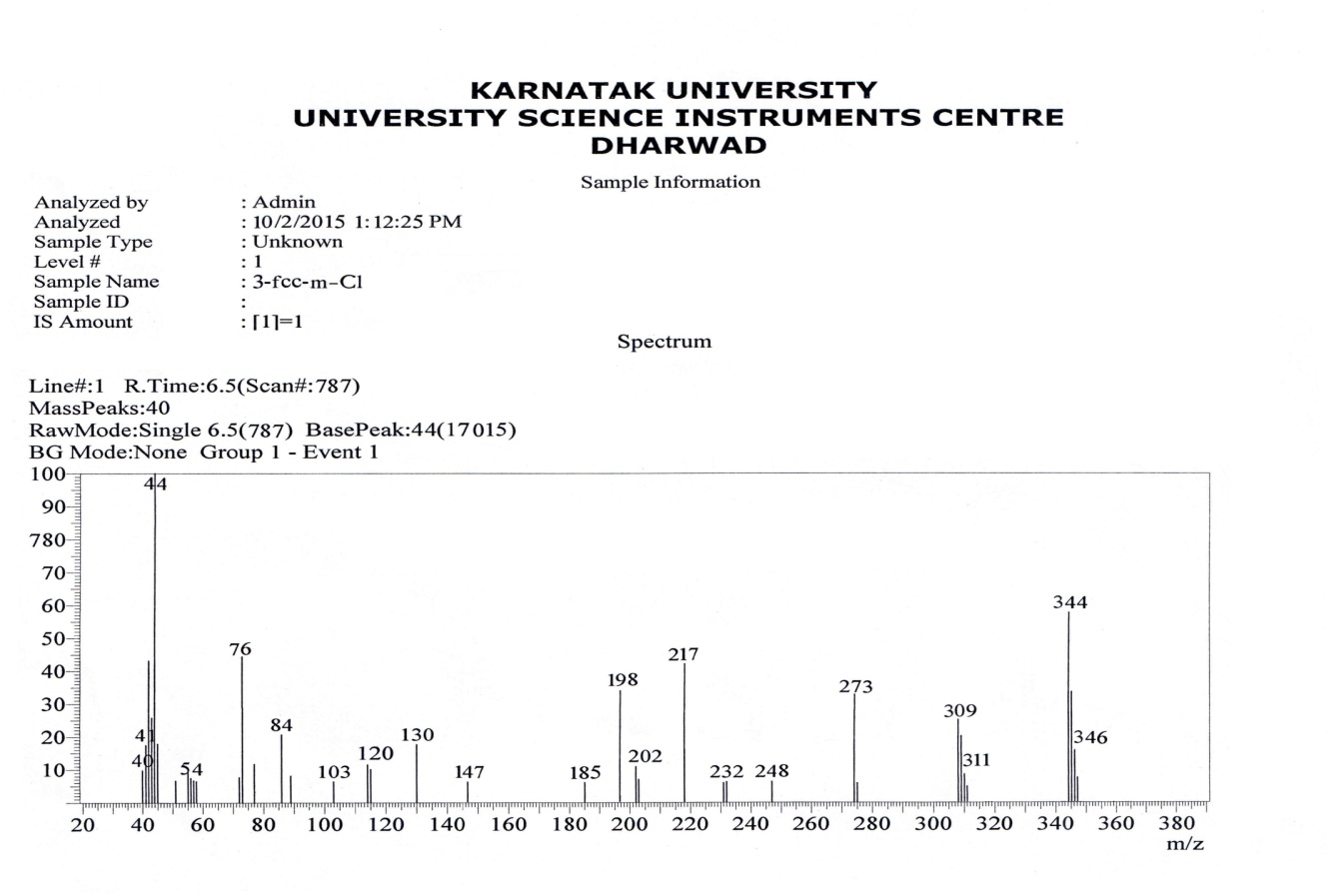


Mass Spectra of **1h**

IR Spectra of **1
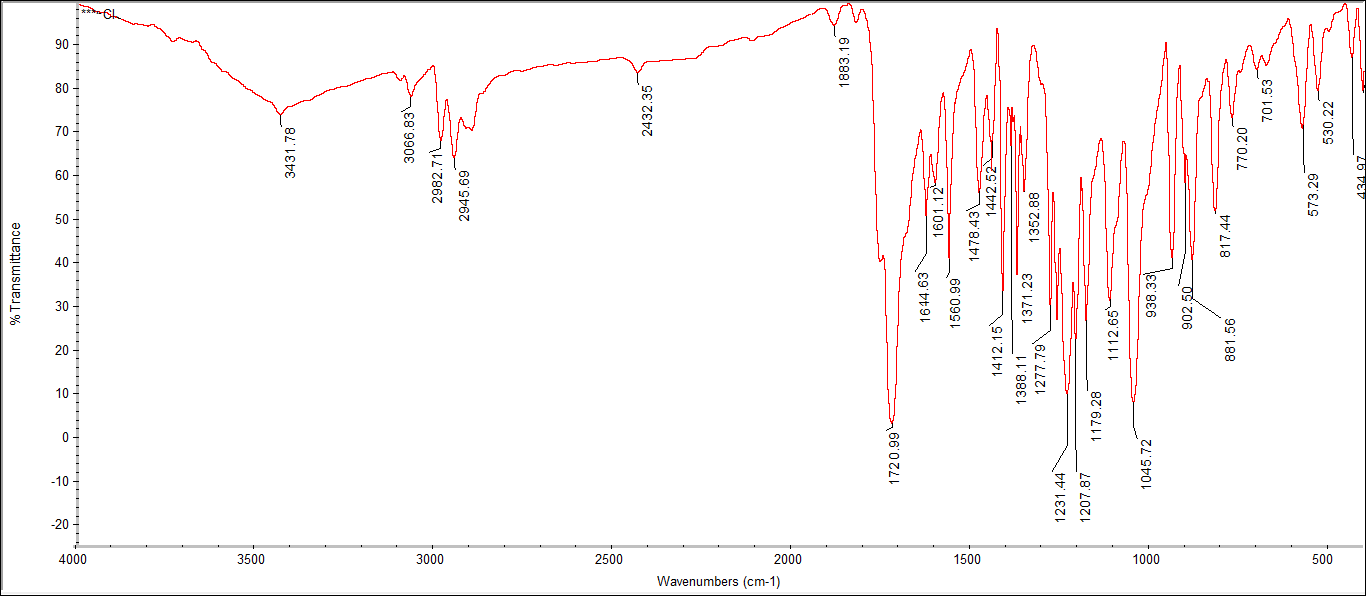
i**


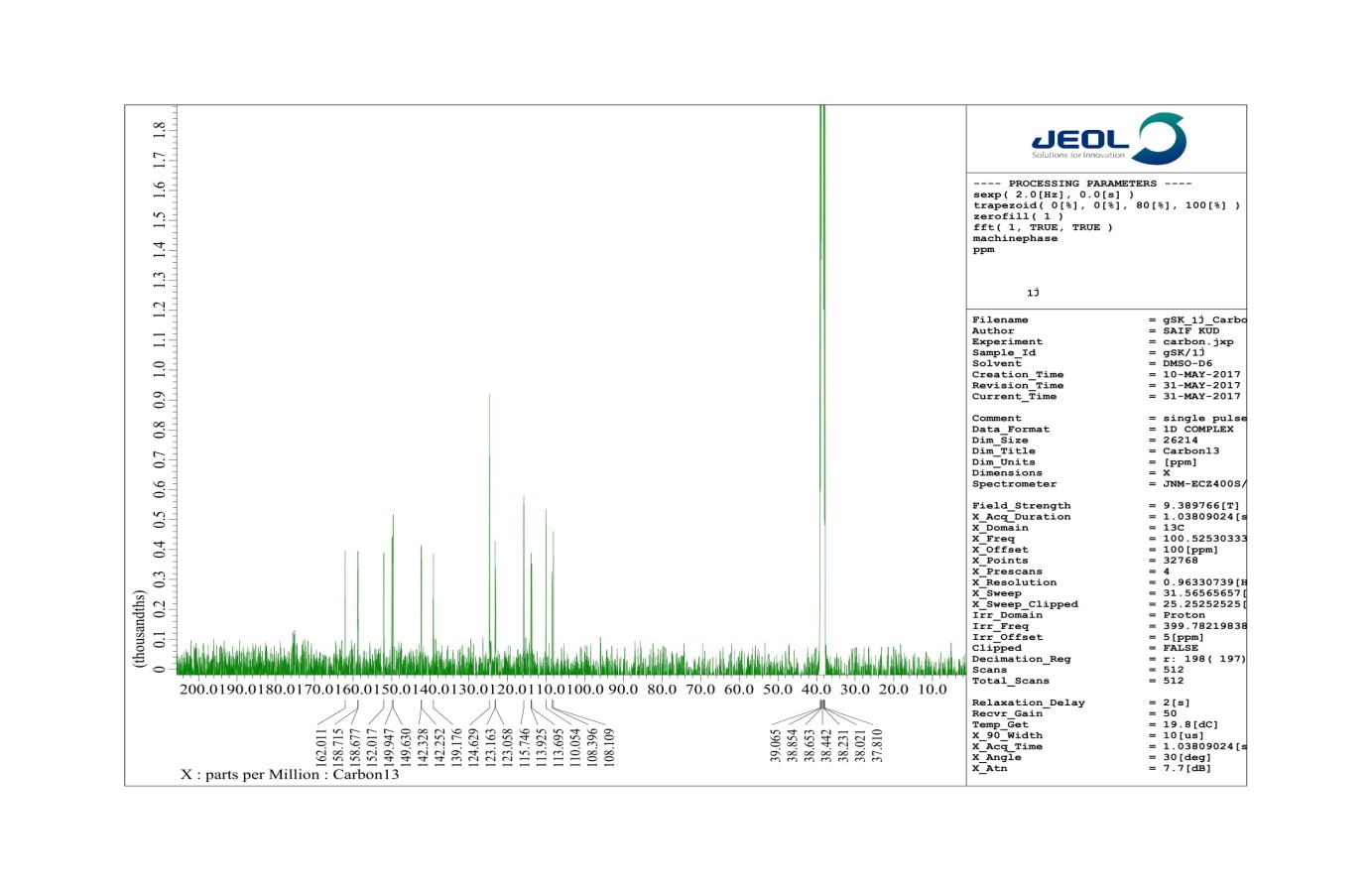


^13^C NMR Spectra of **1i**


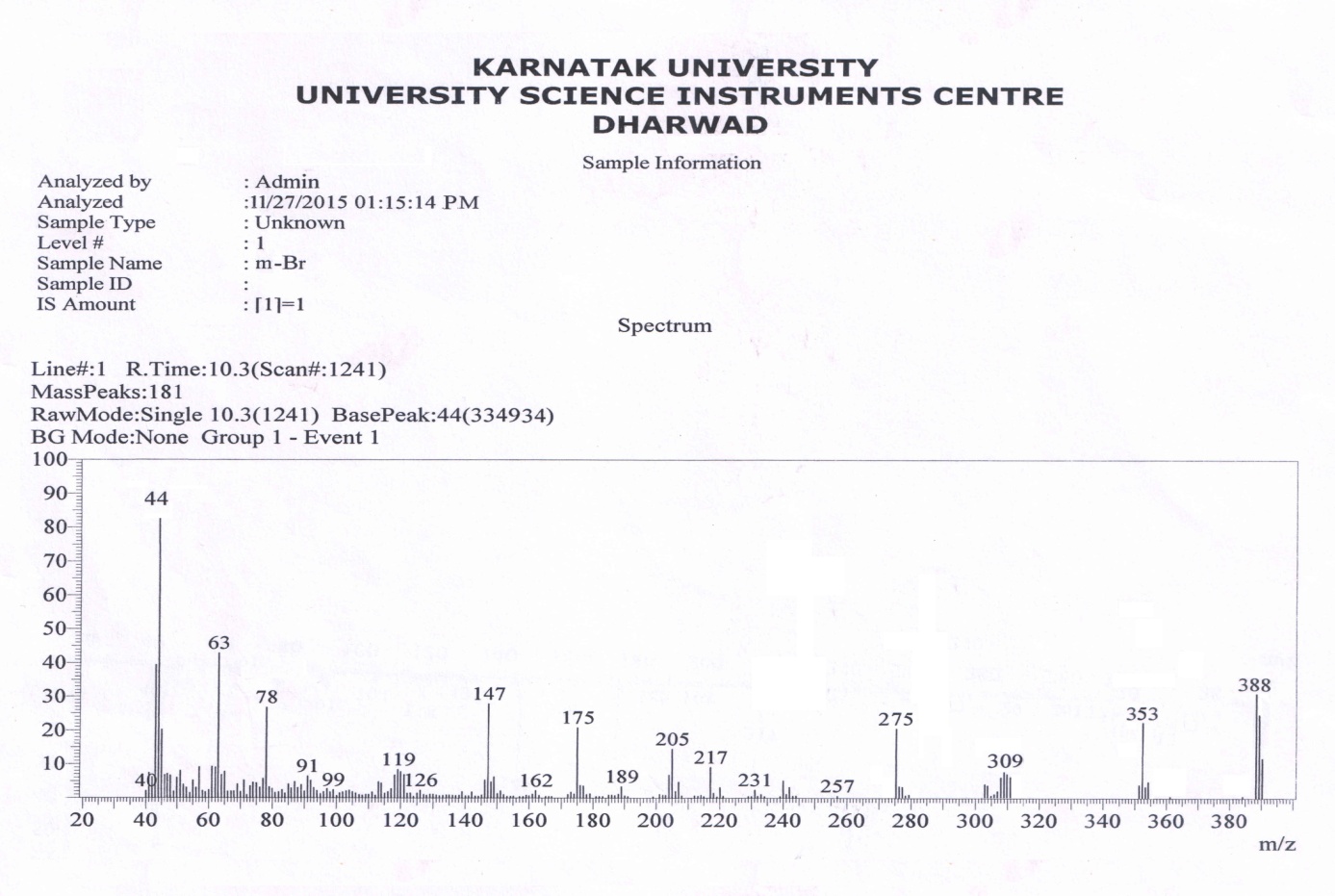


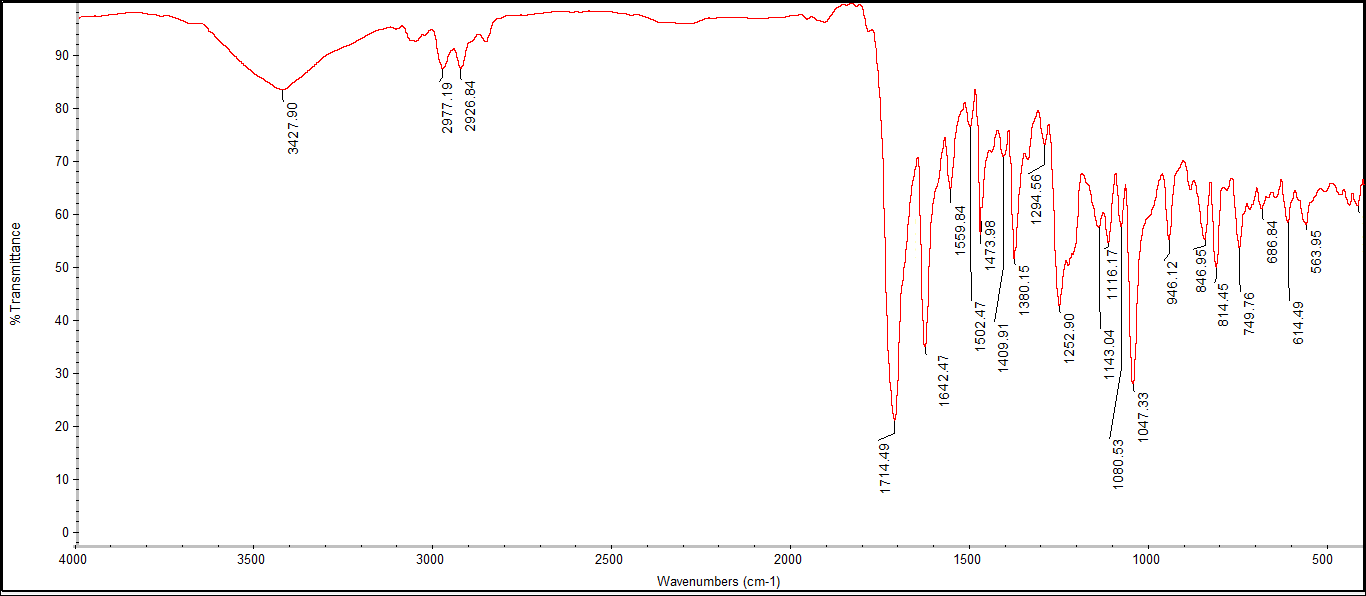
Mass Spectra of **1i**

IR Spectra of **1j**


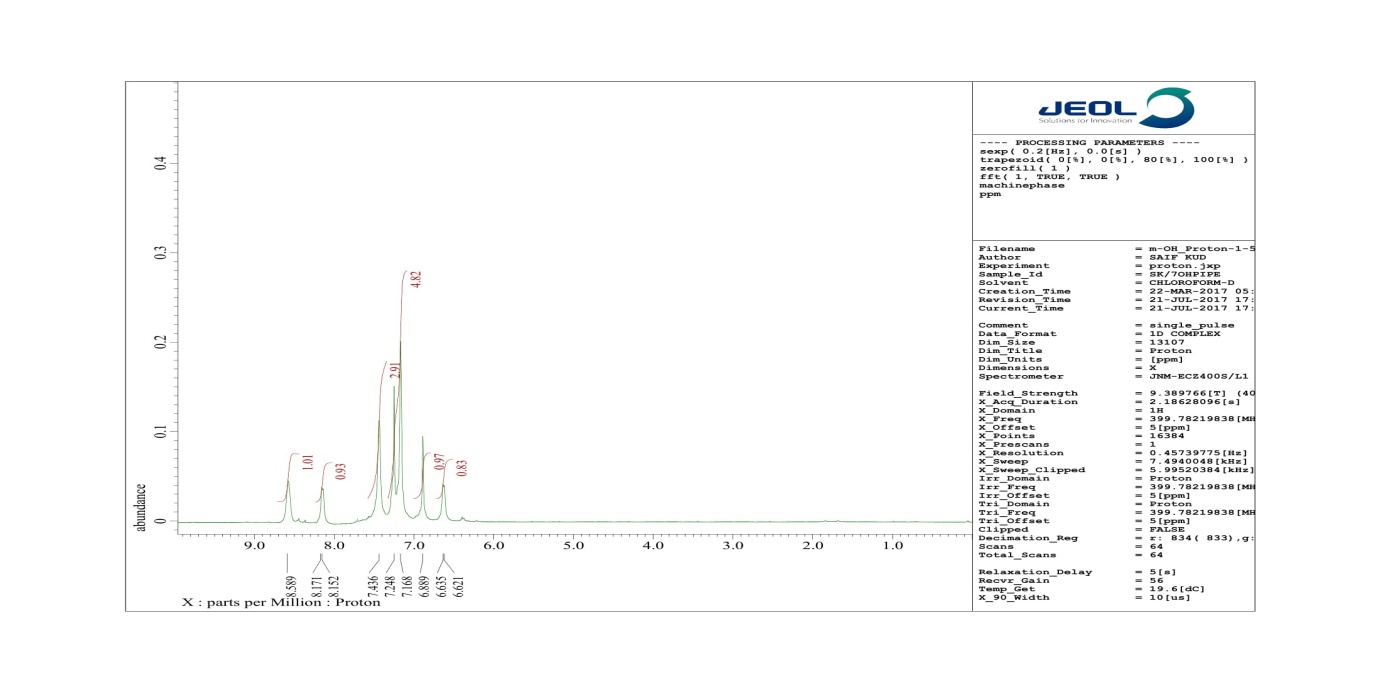


^1^H NMR Spectra of **1j**


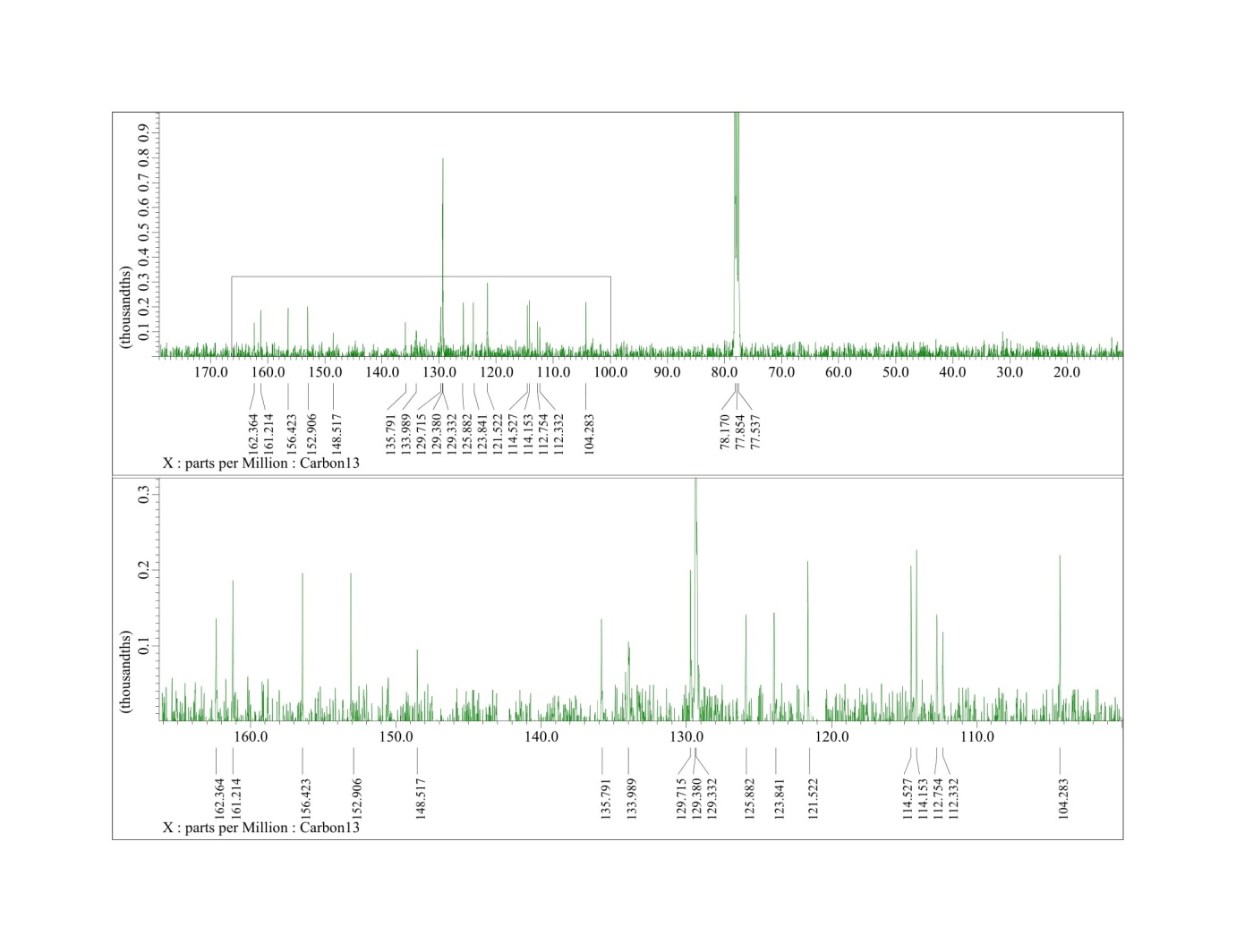


^13^C NMR Spectra of **1j**

Mass Spectra of **1
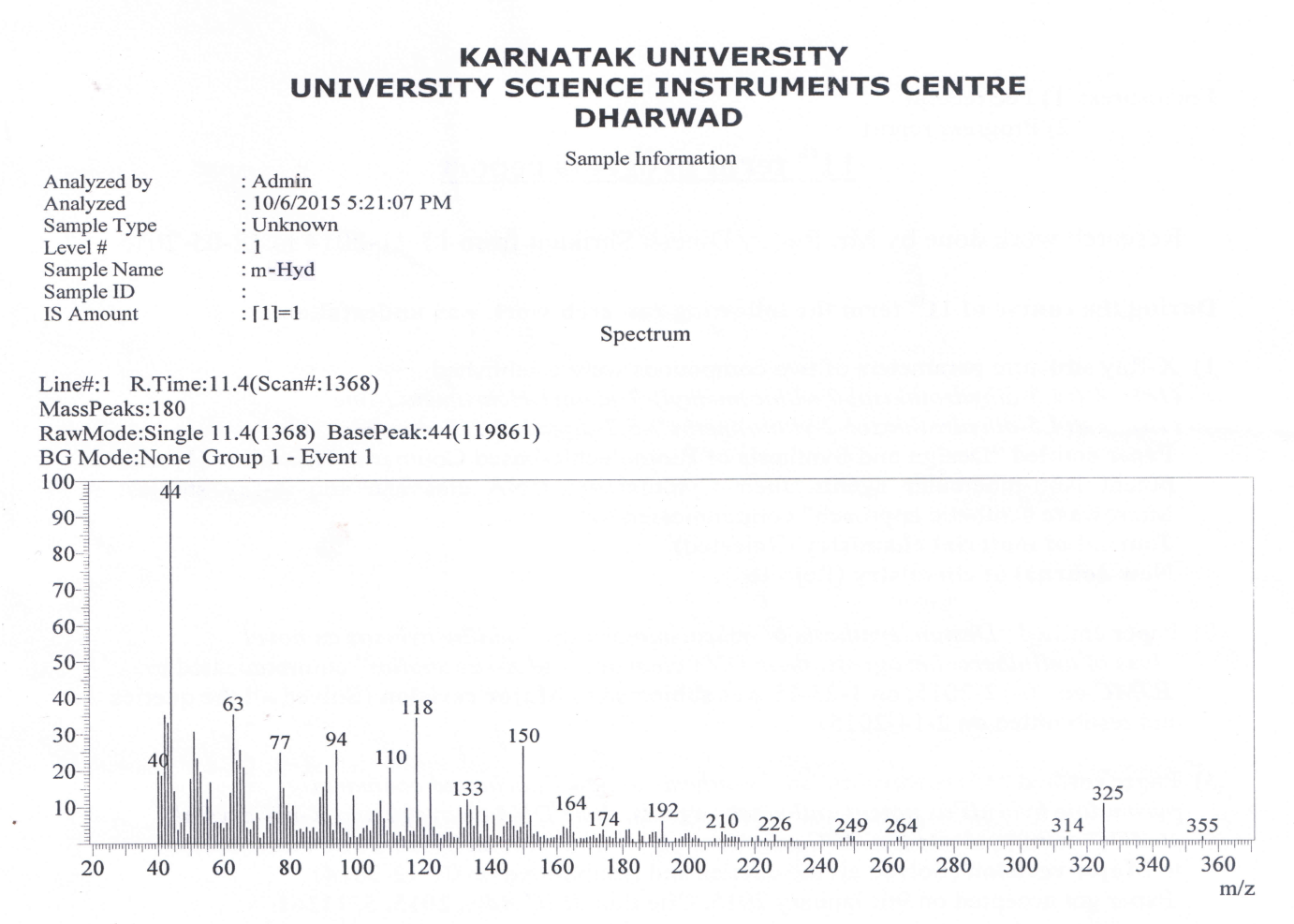
j**


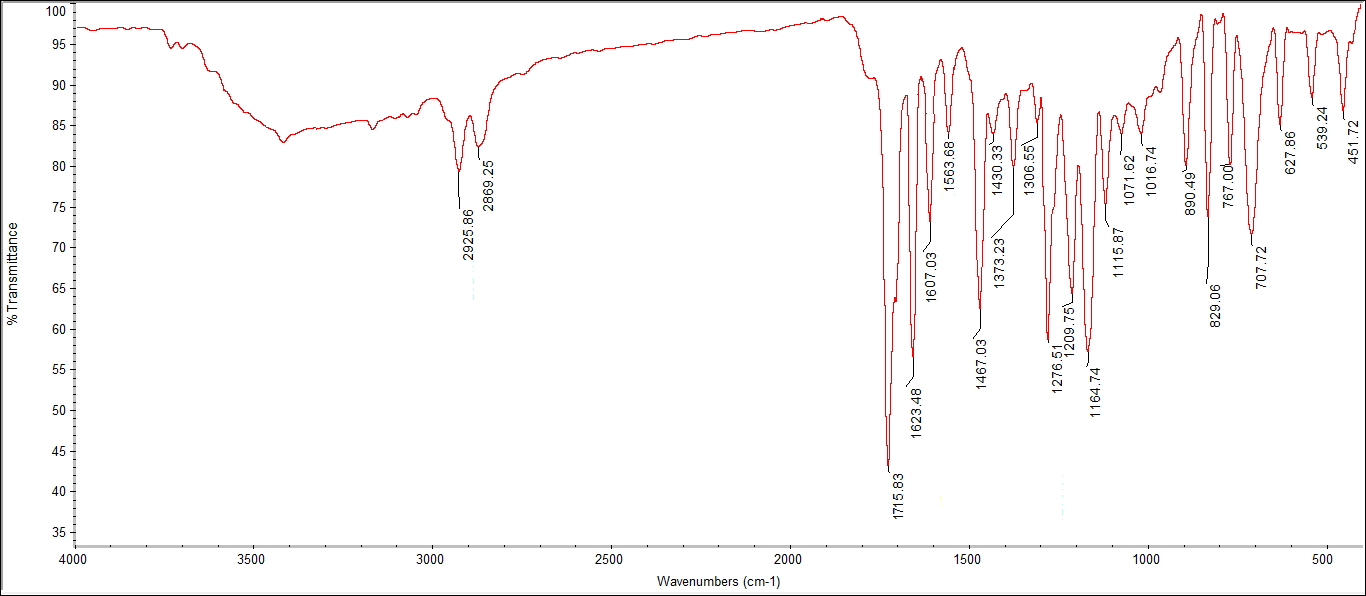


IR Spectra of **1k**

^1^H NMR Spectra of **1
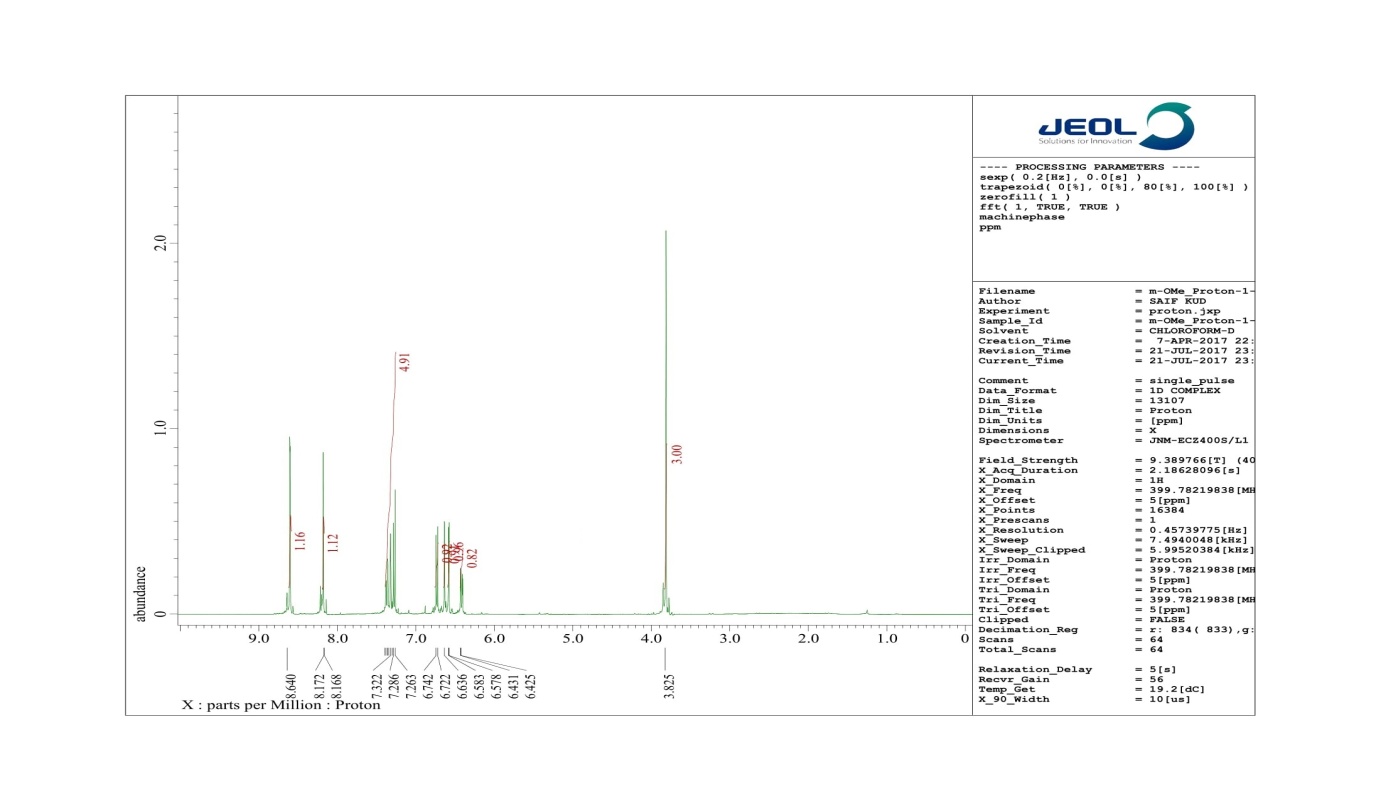
k**


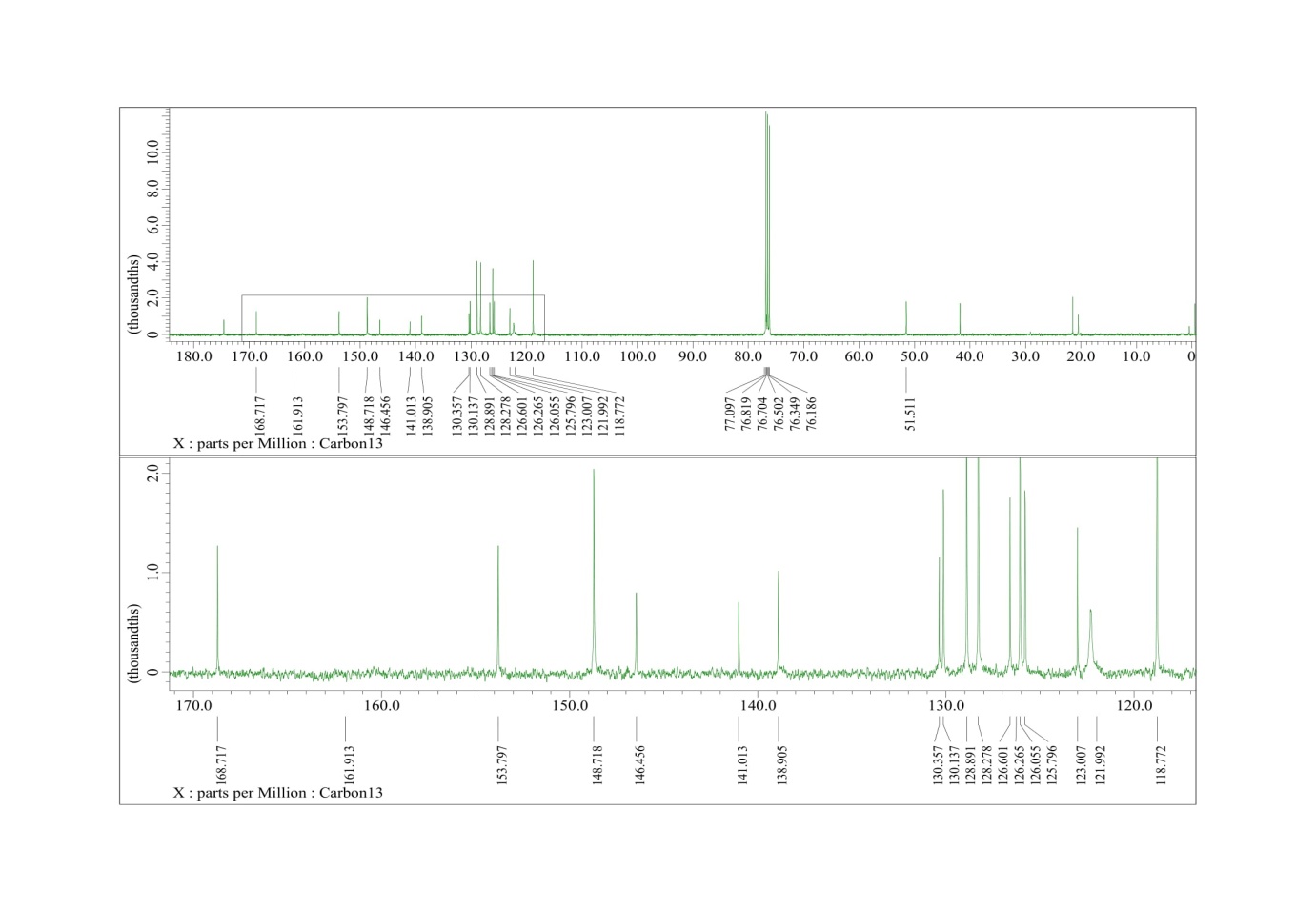


^13^C NMR Spectra of **1k**


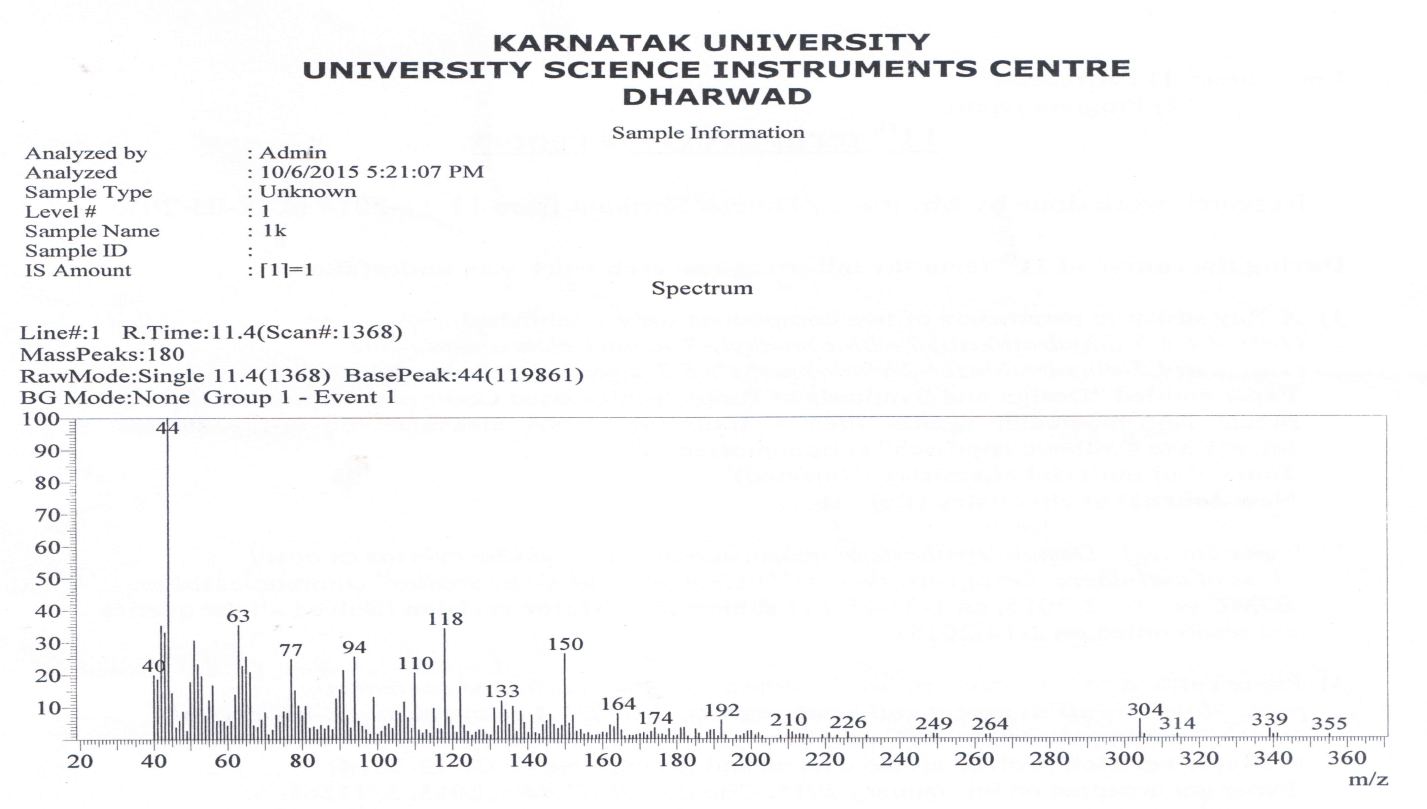


Mass Spectra of **1k**


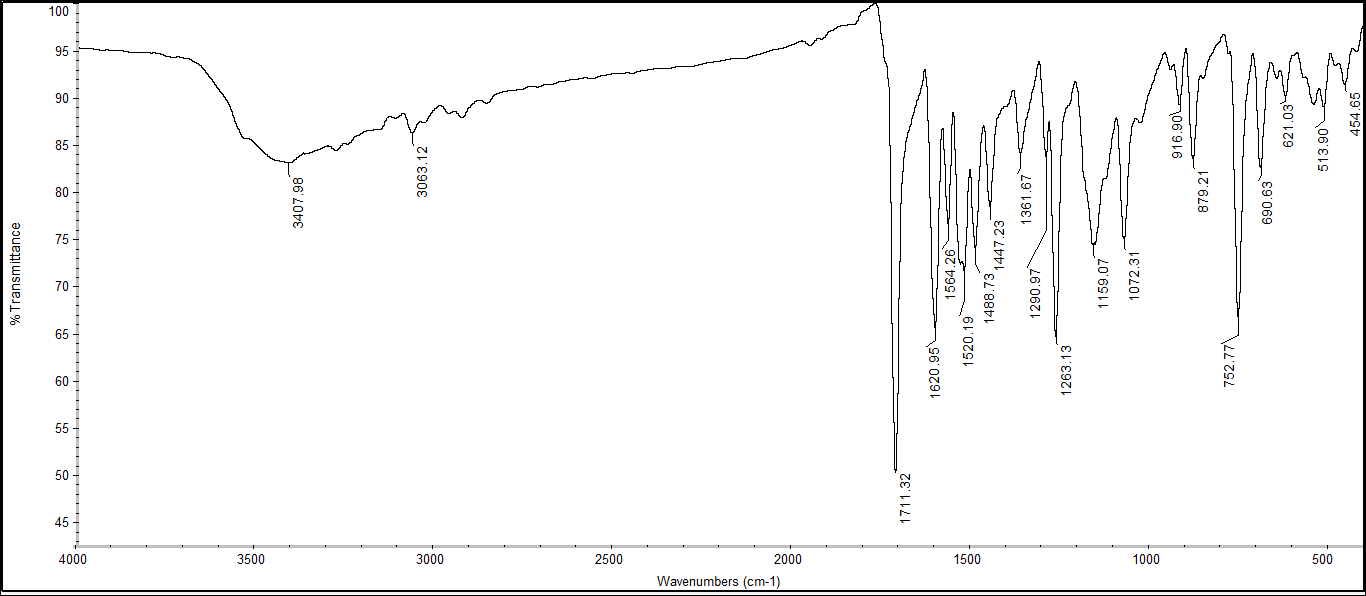


IR Spectra of **1l**

^
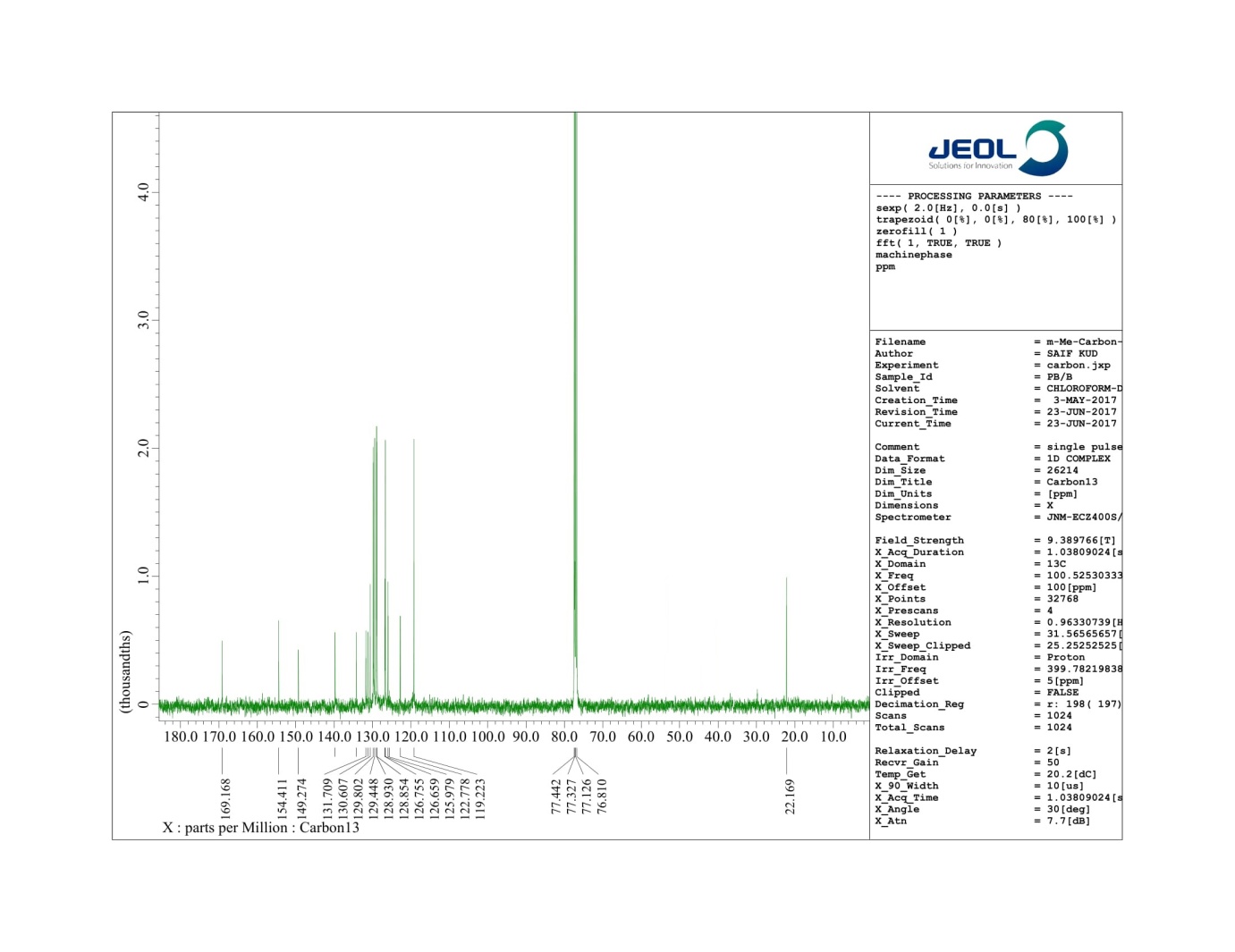
13^C NMR Spectra of **1l**
